# Evidence for coordinate CTCF and histone H3.3 activities in K27M diffuse midline gliomas

**DOI:** 10.1186/s40478-026-02290-2

**Published:** 2026-05-02

**Authors:** Rachel H. Klein, Jennifer Q. Yee, Paul S. Knoepfler

**Affiliations:** Department of Cell Biology and Human Anatomy, University of California, Davis, CA 95616 USA

**Keywords:** Diffuse midline glioma, CTCF, Histone H3.3, K27M, Chromatin, Bivalent genes, H3K4me3 broad domains

## Abstract

Up to 80% of diffuse midline gliomas (DMGs) are characterized by a lysine to methionine driver mutation (K27M) in the tail of histone variant H3.3, pointing to likely roles for epigenetic mechanisms in K27M-driven tumorigenesis. Understanding the effects of mutant histone H3.3 on the complex patterns of histone modifications and interactions with chromatin structure and modifying enzymes is essential to developing effective combination treatment therapies for K27M DMG such as targeting multiple epigenetic enzymes at once. Here, using a genomics approach, we identified combinatorial patterns of epigenetic modifications that are affected by mutant H3.3 in DMG. We also characterized a strong association between H3.3 and the structural chromatin regulator CTCF, finding that mutant H3.3 leads to ectopic binding of CTCF at many additional sites across the genome in DMG. Notably, a number of these ectopic CTCF binding events occur within the *HOX* gene loci and are associated with an increase in H3K27me3 levels at bivalent domains and a decrease in *HOX* gene expression. We also find an association of H3.3 and CTCF at genomic sites adjacent to regions with active or repressive modifications, suggesting a potential role for these two factors in segmenting the chromatin and regulating, perhaps insulating, different types of domains. Together our data suggest that H3.3 K27M both affects epigenetic marks and chromatin organization in part through interaction with CTCF and point to a potentially novel contributory role for CTCF in promoting oncogenesis in DMG. These findings could have potential implications for designing therapy regimens to more effectively target the chromatin changes and genomic instability observed in H3.3K27M glioma cells.

**Supplementary Information:**

The online version contains supplementary material available at 10.1186/s40478-026-02290-2.

## Introduction

High-grade pediatric gliomas are a leading cause of cancer deaths in children. Among these, high-grade diffuse midline gliomas (DMG) have a particularly poor prognosis, with a 2-year survival rate of less than 10% [17]. A unique hallmark of this tumor type is a lysine to methionine mutation in the tail of histone variant H3.3 (H3.3K27M), which is found in approximately 80% of DMG in the pediatric population, but is rare in adult tumors [9, 26, 50, 56]. K27M can act as a cancer driver mutation, causing genomic instability and large-scale changes in epigenetic modifications and chromatin domains [4, 5, 18, 19], but many open questions remain about how it drives gliomagenesis through altering the epigenome. K27M likely also functionally interacts with a core group of other mutations found in these DMG including in TP53 [52, 60].

In a normal context, histone H3.3 appears to play a dual role in chromatin regulation. It is associated with active chromatin at enhancers and promoters, but also with repressed heterochromatic domains [27]. The mechanisms that allow it to carry out such different functions are likely context specific and depend on distinct chaperones (HIRA and DAXX) but have not been fully characterized [11]. Histone H3.3 is also unusual among H3 family members in that it is encoded by *H3F3A* and *H3F3B* genes outside of the H3 gene clusters. In addition, H3.3 can be deposited in chromatin throughout the cell cycle, unlike the canonical versions of H3 [27]. It may be in part due to this replication-independent deposition property that histone variant H3.3 plays an outsized role in chromatin structure and regulation in the developing brain. Indeed, despite being one of the more than 30 histone H3 family genes in the human genome, the lysine to methionine mutation usually found in a single allele of *H3F3A* results in a significant global reduction in H3K27me3, an increase in H3K27ac, and DNA hypomethylation, among other epigenetic effects in DMG [4, 32], even though some wildtype H3.3 protein is still produced from *H3F3B* and one allele of *H3F3A*. These epigenetic changes are thought to feed into key transcriptomic changes such as in neurodevelopmental genes, suggesting that K27M DMG may in part be a neurodevelopmental cancer. Several lines of evidence point to H3.3 playing key normal roles in brain development and function as well [6, 7, 14, 27].

Due to the strong effect of the H3.3K27M mutation on chromatin modifications in DMG, numerous epigenetic drugs have been trialed to treat these tumors including demethylase inhibitors like GSKJ4 [24, 40], HDAC inhibitors such as panobinostat [21], the BET inhibitor JQ1 [37, 46], and DNA demethylase inhibitors [28]. Each has shown some effectiveness in altering the epigenetic landscape and reducing tumor cell growth, but all trialed drugs have also been found to have limitations as therapeutic options including the development of resistance. Several studies have tried combinations of epigenetic drugs and demonstrated higher efficacy against tumor growth, suggesting that targeting multiple epigenetic pathways may yield better results in clinical trials [16, 18, 37]. This makes a great deal of sense, as chromatin domains are often defined by a combination of epigenetic marks and there is a complex interplay between the different modifications, the reader and writer proteins that bind these marks, and the associated factors regulating chromatin structure. A more detailed definition and characterization of these domains can help advance understanding of the chromatin states and regulatory dynamics at play in DMG, point to tumor dependencies, and potentially suggest combinations of drugs that could act most effectively on multiple types of chromatin domains for clinical benefit.

In addition to the composition of chromatin domains, maintaining boundaries between active and repressed regions and facilitating contacts between different classes of domains like enhancers and promoters is crucial to preserving proper chromatin structure and gene expression patterns. CCCTC-binding factor or CTCF is a multifunctional and ubiquitously expressed zinc finger protein that binds DNA and acts as an insulator between different types of chromatin domains, while also having roles in chromatin structure and looping between promoters and regulatory regions that impact gene expression [38, 43, 45]. Disruption of CTCF function and associated changes in the structure of chromatin domains are often found in cancer. Indeed, mice with only one functional copy of *CTCF* show increased incidence of cancer [25], and tumorigenesis is sometimes associated with changes in CTCF binding events [13], often linked to oncogenic alterations in transcription factor binding.

In this study we identified and characterized key chromatin domains in DMG cells, looking at well-known types of domains including super enhancers, bivalent domains, and H3K4me3 broad domains as well as the predicted chromatin states identified by ChromHMM. In this context, we identified a key role for CTCF in maintaining H3K27me3 levels at bivalent domains in DMG, and demonstrated that the H3.3K27M mutation affects both CTCF localization and H3K27me3 at these regions. We further characterized a functional interaction between H3.3 and CTCF in DMG cells, finding that the H3.3K27M mutation leads to an expansion of CTCF-bound domains including a large number of ectopic CTCF binding sites found in H3.3K27M but not WT DMG cells. We also found that even modest CTCF knockdown affected the expression of a set of genes highly enriched in active chromatin states that could be responsible for some of the oncogenic properties of these cells. Together, our results identified complex chromatin domains in DMG defined by combinations of epigenetic modifications, which may have implications for designing therapy regimens that include multiple epigenetic drugs to more effectively target the numerous changes in chromatin observed in glioma cells with H3.3K27M. Our work also suggests roles for the structural protein and transcription factor CTCF in influencing the epigenetic landscape and transcriptome in the context of the H3.3K27M mutation in DMG cells.

## Materials and methods

### Cell culture

Patient-derived pediatric glioma cells (SU-DIPG-XIII and SU-DIPG-XVII) were generously provided by Dr. Michelle Monje. SU-DIPG-XIII H3.3WT were previously generated by gene editing from SU-DIPG-XIII [10]. Cells were grown in Tumor Stem Medium, which contains DMEM/F12 1:1 (Invitrogen), Neurobasal-A (Invitrogen), 10mM HEPES (Invitrogen), 1X MEM sodium pyruvate (Invitrogen), 1X MEM nonessential amino acids (Invitrogen), 1% GlutaMax (Invitrogen), human-bFGF (20 ng/ml) (Shenandoah), human-EGF (20 ng/ml) (Shenandoah), human PDGF-A and PDGF-B (20 ng/ml) (Shenandoah), heparin (10 ng/ml) (StemCell Technologies) and B27 without Vitamin A (Invitrogen) [10].

### Transfection/ siRNA

Cells were seeded onto laminin-coated plates and transfected the following day with the indicated amounts of either scramble control (Qiagen 1027280) or pooled CTCF siRNAs (Dharmacon L-020165-00-0005) using the RNAi Max reagent (Invitrogen 13778075) and OptiMEM medium (Gibco 31985070). Samples were collected 72 h after transfection. A different CTCF siRNA (Thermo Fisher AM16708) was used to replicate the results of cell viability and cell count assays.

### Cell viability assay

Cells were seeded on laminin-coated plates and transfected with CTCF or scramble control siRNA. Cells were collected with TrypLE 72 h after transfection and used for the CellTiter Glo Luminescent cell viability assay (Promega G7570). After addition of CellTiter glo reagent, plates were read on the SpectraMax^®^ i3x Multi-Mode Microplate Reader (Molecular Devices) with SoftMax^®^ Pro 7 Software.

### Cell counts

Cells were collected using TrypLE (Gibco 12604013) 72 h after transfection. They were then washed with PBS, placed on ice and stained with propidium iodide (EMD Millipore Corp 537059-50MG) to stain dead cells on the same day. Positive controls were treated with 70% ethanol while negative controls were unstained live cells. These were used to create the gate for the PI staining. Flow cytometry was performed using the Attune™ NxT Acoustic Focusing Cytometer (Thermo Fisher Scientific). Data was acquired and analyzed using Attune™ NxT Software (version 3.1.2).

### Western blotting

Nuclear extraction was performed using the Abcam nuclear fractionation protocol. Briefly, cells were resuspended in 10 mM HEPES, 1.4 mM MgCl2, 10 mM KCl, 0.5 mM DTT and 0.05% NP40 at pH 7.9, then were centrifuged. The resulting pellet was resuspended in 5 mM HEPES, 1.5 mM MgCl2, 0.2 mM EDTA, 0.5 mM DTT, 26% glycerol (v/v) and 4.6 M NaCl at pH 7.9 and the supernatant collected after centrifugation. After boiling for 10 min with DTT, samples were run on NuPAGE Bis-Tris mini gels (Novex) and membrane transfer was performed in 1X MES NuPAGE buffer (Novex). After blocking, blots were incubated with primary antibody overnight (CTCF Millipore #07-729, mouse anti-H3 Upstate/EMD-Millipore, #05-499, rabbit anti-H3K4me3 Cell Signaling 9751I). Blots were incubated with goat anti-mouse (1:10,000 dilution IR Dye 680RD, LiCOR 926-68070) or goat anti-rabbit secondary antibodies (1:10,000 dilution IR Dye 800CW, LiCOR 926–3211) for 1 hour at room temperature and imaged using the Odyssey cLX Imaging system (LiCOR).

### Chromatin Immunoprecipitation (ChIP)

ChIP was performed as described previously [41]. Briefly, cells were crosslinked with 1% formaldehyde, lysed, and sonicated using a Bioruptor Pico (Diagenode) to generate chromatin fragments < 500 bp. For each ChIP, 20–30ug of sonicated chromatin was used and immunoprecipitated on magnetic Dynabeads (Pierce #88802). The following antibodies were used: IgG (Santa Cruz, #sc-2027), rabbit anti-H3.3 (Millipore #09-838), rabbit anti-CTCF (Millipore #07-729), and rabbit anti-H3K4me3 (Cell Signaling 9751I). Antibodies used for ChIP were validated using ChIP-qPCR to demonstrate enrichment at predicted binding sites relative to negative control regions (Supplemental Figs. 1b, 6a, 9b).

### ChIP-seq

Libraries were prepared using the NEB Next Ultra II DNA Library Prep Kit and sequencing was performed on the Novaseq 6000 platform. ChIP-Seq reads were aligned to the hg19 genome using Bowtie2 [29, 30]. MACS2 was used to call peaks, with an input sample used as the background control and an FDR cutoff of 0.05 [61].

### CUT&RUN

CUT&RUN was performed for CTCF (rabbit anti-CTCF, Millipore #07-729) using the ActiveMotif CUT&RUN kit (ActiveMotif #53180) according to the protocol. An Input sample was used as the control. Libraries were prepared using the NEB Next Ultra II DNA Library Prep Kit and sequencing was performed on the Novaseq 6000 platform. Resulting reads were trimmed of adapters with bbduk (bbmap) and were aligned to the hg19 genome with Bowtie2 [29]. Peaks were called with SEACR using the stringent option [36].

### RNA-seq

RNA was collected 72 h after transfection for cells transfected with either scramble control siRNA (Qiagen 1027280) or pooled CTCF siRNA (Dharmacon L-020165-00-0005). There were 2 replicates of each transfection. RNA was extracted using the NucleoSpin RNA extraction kit (Machery Nagel 740955.50). Knockdown was verified by cDNA synthesis and qPCR. Poly-A + non-Directional libraries were prepared and samples were sequenced on the Illumina NovaSeq × Plus with 150 base paired-end sequencing. After a QC check with FastQC [2] and adapter trimming with bbduk (bbmap) [8], transcripts were quantified using Salmon [44] and differential expression analysis was performed using EdgeR [48].

### Sequential ChIP

Two samples of sequential ChIP were performed for H3.3 (Millipore #09-839) and CTCF (Millipore 07-729-25UL) antibodies: one sample with H3.3 immunoprecipitated first followed by CTCF, the second with CTCF immunoprecipitated first followed by H3.3. Chromatin was immunoprecipitated twice with IgG antibodies as a negative control. Sequential immunoprecipitation with antibodies to H3K27ac and H3K9ac was performed as a positive control. Sequential ChIP was performed as previously described [3] with the following exceptions: 90 ug of chromatin was used for each immunoprecipitation, and protein A/G magnetic beads (Pierce #88802) were used to pull down protein/DNA complexes. After elution and reversal of crosslinking, resulting DNA was purified using the Qiagen PCR clean up kit. qPCR was run on the resulting DNA to determine enrichment at predicted H3.3 and CTCF co-bound sites.

### Quantitative PCR (qPCR)

ChIP’ed DNA was diluted 1:4 and used in qPCR reactions with PowerUp SYBR Green Master Mix (Applied Biosystems A25742). Primer sequences are listed below:

### ChIP primer sequences

**Table:** 

ASCL1 ChIP F	TCTAAGAAGTCTCCCGGGGA
ASCL1 ChIP R	GAACTTGGGTGCAGGAACAG
HES5 ChIP F	CCACCTAGTCTCTCTGGCAG
HES5 ChIP R	GATCCTCAAGTCTGCCACCT
OLIG1 ChIP F	CAGAAAGTGCTCGCTCTCAC
OLIG1 ChIP R	AGGAAAAGAACCACCCCTCC
OLIG2 ChIP F	CGTCTCAAGATCAACAGCCG
OLIG2 ChIP R	CGTAGATCTCGCTCACCAGT
COL20A1 ChIP F	GAGTGGGATCAGGAGCAGAG
COL20A1 ChIP R	ACCTGGCTTCTCTTCTGTCC
ChIP-IL5-neg F	TCAGCAGAGTTCGATGAGTAGA
ChIP-IL5-neg R	GCAGAACGTTTCAGAGCCAT
NAV1 ChIP F	CGCAGAGCTGTTCCATTGTT
NAV1 ChIP R hChIP Hoxa9 F hChIP Hoxa9 R hChIP Hoxa7 F hChIP Hoxa7 R hChIP Hoxc5 F hChIP Hoxc5 R hChIP Hoxd F hChIP Hoxd R	CCGGCACATCCCAGATCTAT TAGGGCGGCTGTTCACTAAA CTGAGGCTGCAGTACCAAAC GCCGTCCTTCTTTGCCATAG CTCTGGCTTTGGGATTCTGC TTCCATCACTAACCTCCCGG TCCTATGCGCTCTTCCCAAA AATACGAGACGAGGGCCAAA CTGAAGTGTGGCGGTTTGAA

### qPCR primer sequences

**Table:** 

GAPDH	Forward	GGAGCGAGATCCCTCCAAAAT
GAPDH	Reverse	GGCTGTTGTCATACTTCTCATGG
CTCF	Forward	CAGTGGAGAATTGGTTCGGCA
CTCF	Reverse	CTGGCGTAATCGCACATGGA

### Super enhancers/H3K4me3 broad domains

Super enhancers in Line XIII were previously defined [37]. Briefly, peaks were called from H3K27ac data and enhancers identified as H3K27ac peaks at least 2 kb away from transcriptional start sites. Super enhancers were identified by stitching together enhancers withing 12.kb of each other and ranking by H3K27ac read density relative to input control using ROSE [35]. H3K4me3 broad peaks were called with MACS2 using the broad peak (-broad) option [61]. Resulting peaks were ranked by size and the top 5% of peaks were identified as H3K4me3 broad peaks.

### Enrichment analysis

To assess whether the overlaps between two types of genomic regions were greater or less than expected by chance, we used a permutation-based empirical approach. For each pair of BED files, the observed number of overlapping intervals was computed using *bedtools intersect* with the -wa -u options. A null distribution was generated by randomly shuffling the primary BED intervals across the genome (*bedtools shuffle*) excluding blacklisted regions and recalculating overlaps with the comparison BED file. *P*-values were computed as the fraction of shuffled overlaps greater than or equal to the observed value, with pseudocount correction.

### Motif analysis

Motif analysis was performed using Homer’s findMotifsGenome.pl tool [20] with the -size 200 option [20]. Due to the large size of super enhancers and H3K4me3 broad domains, we focused the motif enrichment analysis on regions of open chromatin within these domains.

### ChromHMM

Data from Line XIII H3.3K27M cells were collected from GEO and analyzed. ChromHMM [12] was used to identify chromatin domains in Line XIII cells with the following datasets: H3K27me3 [10], H3.3 [10], H3K4me3 (this paper), H3K4me1 [1], H3K4me2 [23], H3K27ac [37], H3K36me2 [59], H3K27me1 [19], H3K27me2 [19], ATAC-seq [33], and DNA methylation [34]. The number of domains to find was set to 12.

### Partially methylated domains

Sequencing reads were aligned to the hg19 genome using BSMAPz [57], and partially methylated domains were identified using the methratio.py function within BSMAPz.

### Diffbind

Differential peak analysis between Line XIII H3.3WT and Line XIII H3.3K27 mutant cell lines was performed with the R package DiffBind [49].

### GO analysis

Chromatin domains were overlapped with gene coordinates using BedTools [47] and the resulting list of genes was used for gene ontology (GO) analysis with DAVID [22].

### GEO accession numbers

All of the sequencing data generated in this paper can be found under GEO accession numbers: GSE285924, GSE285840, GSE285841.

**Fig. 1:**
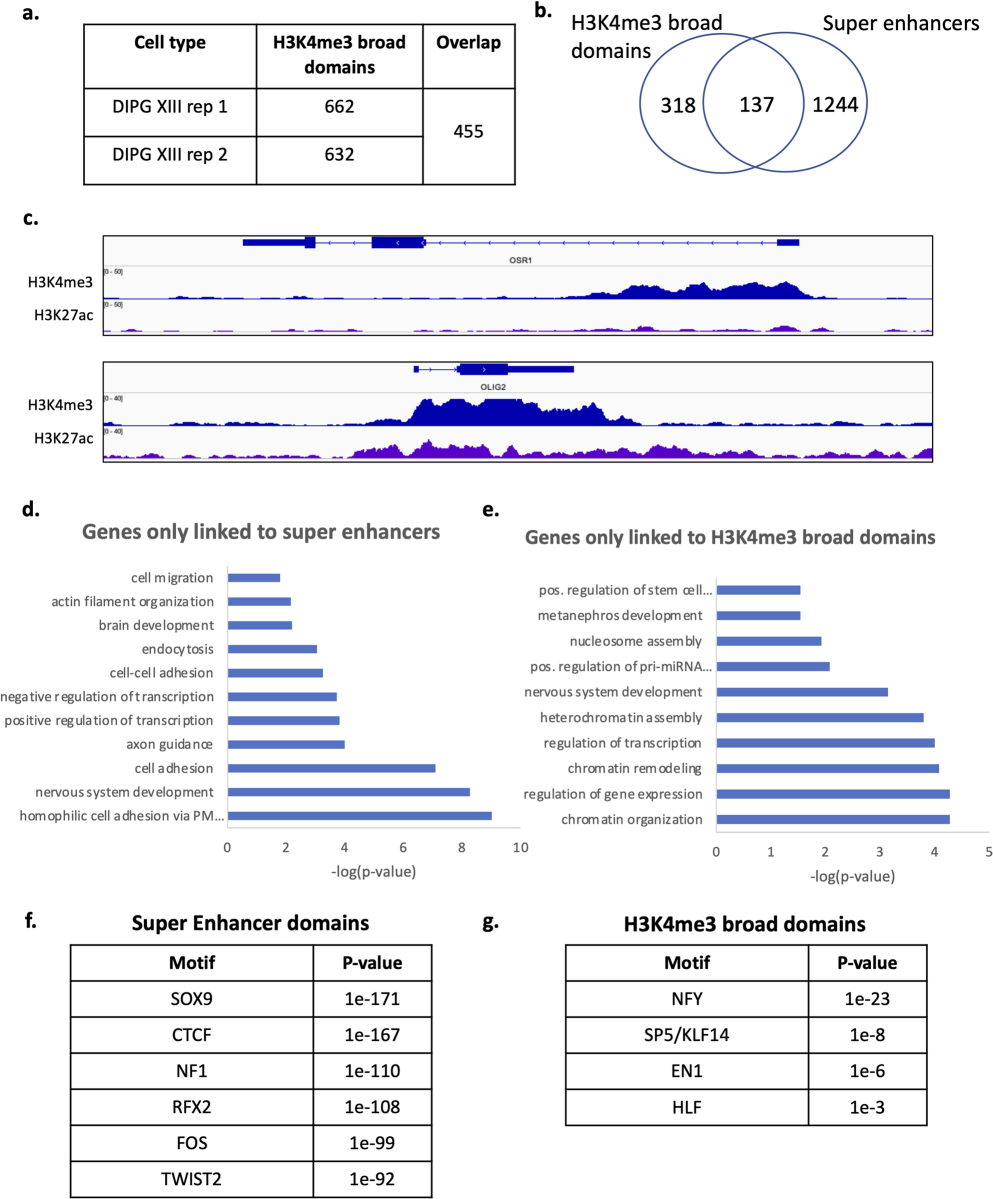
H3K4me3 broad domains overlap key transcriptional regulators and appear to be functionally distinct from super enhancers in DMG. **a** Number of H3K4me3 broad domains identified in each H3K4me3 replicate and the overlap of broad domains between the two replicates for Line 13 DIPG cells. **b** Overlap of H3K4me3 broad domains found in both H3K4me3 replicates with super enhancers identified in Line 13 DIPG cells by the Monje Lab. **c** Track image of a H3K4me3 broad domain without a super enhancer overlapping gene OSR1, and track image of a H3K4me3 broad domain with a super enhancer overlapping gene OLIG2. **d** Gene ontology analysis for regions with super enhancers without H3K4me3 broad domains. **e** Gene ontology analysis for regions with H3K4me3 broad domains without super enhancers. **f** Enriched motifs in super enhancer regions without H3K4me3 broad domain. **g** Enriched motifs for H3K4me3 broad domains without super enhancers.

## Results

### H3K4me3 ChIP-seq helps define chromatin domains in K27M DMG

To identify the patterning of the H3K4me3 mark and to help define chromatin domains in K27M DMG cells, we performed ChIP-seq in duplicate in SU-DIPG-XIII cells (hereafter referred to as line XIII). We identified 14,745 and 15,811 H3K4me3 peaks in each replicate, and 13,925 peaks were found in both replicates (Supplemental Fig. 1b), indicating a strong concordance between samples. We also found a high overlap between our two H3K4me3 ChIP-seq replicates and a single replicate of published H3K4me3 peaks identified using CUT&RUN (Supplemental Fig. 1b) [15].

As expected, the majority of H3K4me3 peaks were found within 5 kb of gene promoters in Line XIII DMG cells (Supplemental Fig. 1c). Gene ontology analysis revealed that genes with proximal H3K4me3 peaks in DMG are enriched in many general functional categories expected to be active in cycling cells. These include regulation of transcription, cell division, RNA processing, DNA repair, and protein ubiquitination (Supplemental Fig. 1d).

### H3K4me3 broad domains in DMG overlap predicted binding sites of key transcriptional regulators and appear to be functionally distinct from super enhancers

As large chromatin domains like super enhancers and H3K4me3 broad domains have important roles in gene regulation, we next identified H3K4me3 broad domains in Line XIII K27M cells using our ChIP-seq data. A total of 662 and 632 broad domains were called in each replicate, with most (455) overlapping between the 2 replicates (Fig. 1a). We were interested to see how these large regions with high H3K4me3 signal compared to super enhancers, which are known to have high H3K27ac signal. We found that 137 regions have both an H3K4me3 broad domain and a super enhancer, however, the majority of each type of domain were unique (Fig. 1b, c). Supporting the idea that super enhancers and H3K4me3 broad domains primarily regulate distinct categories of genes, gene ontology analysis for genes linked to the 1244 super enhancers that do not overlap H3K4me3 broad domains found enrichment in categories related to cell adhesion, actin cytoskeleton organization, and axon guidance, while in contrast, the 318 H3K4me3 broad domains that do not overlap super enhancers are linked to genes with functional roles in chromatin organization and nucleosome assembly (Fig. 1d, e). Regions with both a super enhancer and an H3K4me3 broad domain are associated with genes involved in proliferation, transcriptional regulation, and nervous system development (Supplemental Fig. 1e).

We next identified open chromatin regions within super enhancers or H3K4me3 broad domains by overlapping these domains with ATAC-seq data in Line XIII K27M cells [33]. Motif analysis within these accessible chromatin domains revealed different enriched motifs for super enhancers and H3K4me3 broad domains. While super enhancers were enriched for SOX9, CTCF, NF1, and FOS motifs, H3K4me3 broad domains were enriched in mostly distinct motifs including those for NFY, KLF14, EN1, and HLF (Fig. 1f, g and Supplemental Table 1). These results suggest that super enhancers and H3K4me3 broad domains are primarily distinct both in terms of the gene expression programs they regulate, and the transcriptional regulators that bind these regions in DMG.

To determine whether these findings are consistent across DMG cell lines, we identified super enhancers and H3K4me3 broad domains in two additional DMG cell lines with H3.3K27M mutation (Line VI and Line XVII from GSE158447) and repeated our analysis. While some super enhancers and H3K4me3 broad domains were found consistently across cell lines, there was also a great deal of individual variation, with unique super enhancers and H3K4me3 broad domains identified in each cell line (Supplemental Fig. 1f). Despite the variation in terms of which individual regions are called as super enhancers and H3K4me3 broad domains in each cell line, the properties of these domains remained consistent across cell lines. Similar to Line XIII, super enhancers and H3K4me3 broad domains are primarily distinct regions in Line VI and Line XVII (Supplemental Fig. 2a, c). Across the three cell lines, genes linked to super enhancers fall into GO categories related to cell adhesion, and cell migration, while genes linked to H3K4me3 broad domains are enriched in categories related to chromatin organization, DNA damage response, and transcription (Fig. 1d, e and Supplemental Fig. 2e-h). Motif analysis for super enhancers and H3K4me3 broad domains in lines VI and XVII showed a similar pattern to Line XIII: super enhancer regions are most strongly enriched for SOX family motifs, CTCF, and FOS2L/JUN motifs, while H3K4me3 broad domains are most strongly enriched for NF1 and KLF motifs (Supplementary Fig. 2b, d and Supplemental Tables 2, 3), providing further evidence that these two types of domains consistently have distinct functions and are regulated in different ways in DMG.

### Bivalent domains have increased H3K27me3 levels in H3.3K27M DMG cells compared to WT isotype controls, including at *HOX* gene clusters

**Fig. 2:**
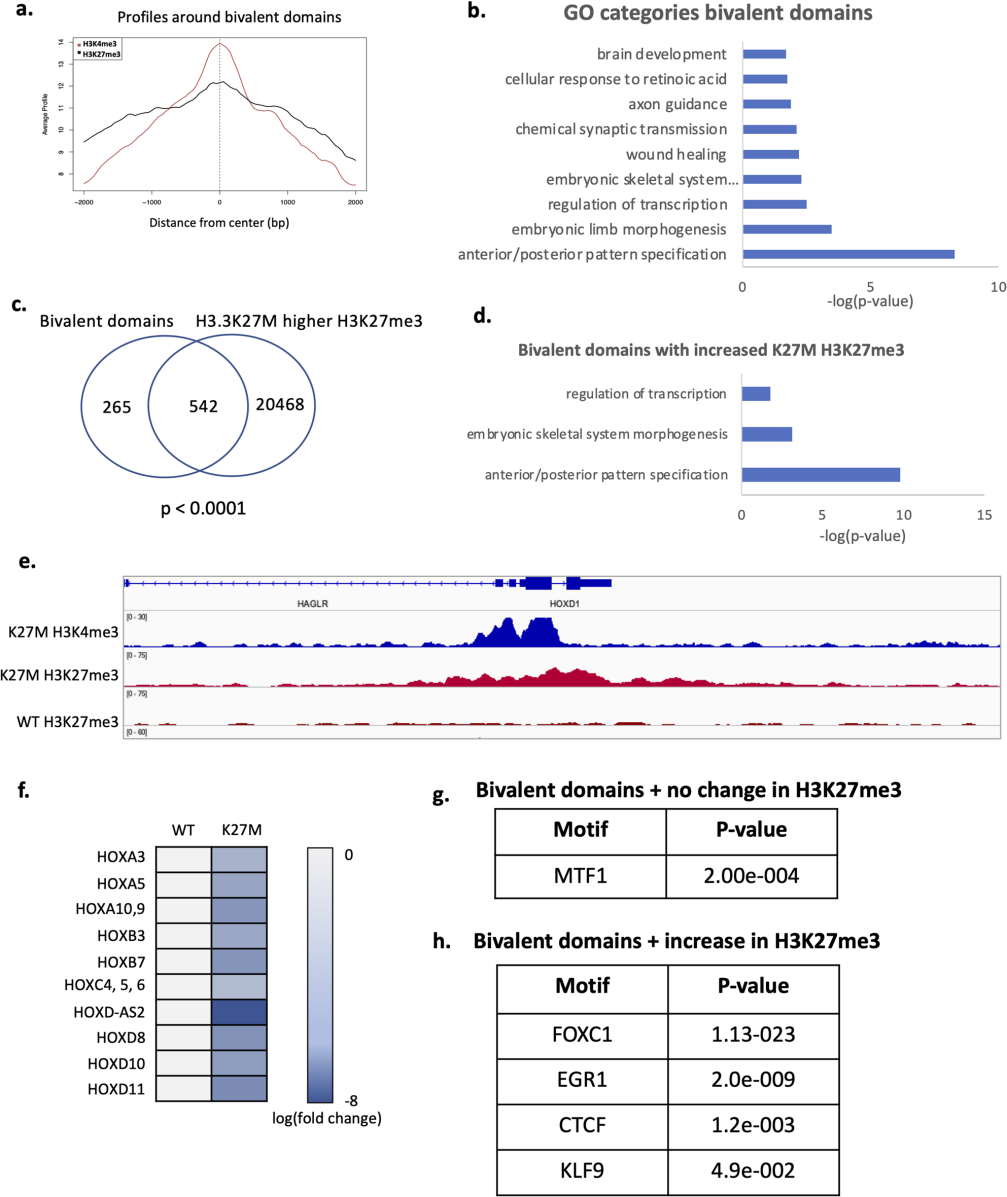
Bivalent domains have increased H3K27me3 levels in H3.3K27M DMG cells compared to WT isogenic controls, including at HOX gene clusters. **a** H3K4me3 and H3K27me3 signal at the identified 807 bivalent domains. **b** Gene ontology of genes associated with bivalent domains in Line XII DMG cells. **c** Overlap of bivalent domains and regions with significantly more H3K27me3 signal in Line XIII cells (H3.3K27M) compared to isotype (WT) controls. **d** Gene ontology analysis for genes associated with bivalent domains with increased H3K27me3 signal in H3.3K27M cells. **e** Track images of bivalent regions with reduced H3K27me3 in H3.3WT cells at HOX gene loci. **f** Changes in HOX gene expression between Line XIII H3.3K27M and WT cells. **g** Enriched motifs in bivalent domains with no change in H3K27me3 in XIII cells. **h** Enriched motifs in bivalent domains with an increase in H3K27me3 in XIII cells.

As combinatorial patterns of chromatin modifications often define functionally distinct chromatin domains, we next looked at how our H3K4me3 data overlapped with other datasets. Using previously published H3K27me3 ChIP-seq data [10], we identified 807 bivalent chromatin regions in Line XIII cells that contain both H3K4me3 and H3K27me3 peaks (Fig. 2a). We also found 1583 such regions in Line XVII, and 3319 in Line VI (Supplemental Fig. 3a-c). Notably, since DMG is thought to be a neurodevelopmental disease, these bivalent domains are near genes involved in functions that include brain development, regulation of transcription, and anterior/posterior pattern specification in all three cell lines (Fig. 2b and Supplemental Fig. 3g, h).

The H3K27me3 mark is known to be affected by the histone H3.3K27M mutation found in a high proportion of DMG tumors, including Line XIII. When Line XIII cells were CRISPR gene edited to revert H3.3 K27M to WT form, approximately 20,000 H3K27me3 peaks were lost, and 80,000 H3K27me3 peaks were gained [10]. To determine whether the H3K27me3 peaks in bivalent domains are affected by the presence of H3.3K27M, we overlapped the bivalent domains with regions that show significant changes in H3K27me3 peaks between the isogenic Line XIII H3.3WT and Line XIII H3.3K27M cells. Surprisingly, the majority of bivalent domains in Line XIII H3.3K27M cells had H3K27me3 peaks that were significantly higher in H3.3K27M cells compared to WT (Fig. 2c, d and Supplemental Figs. 4, 5a, b). This finding was replicated in Line XVII isogenic cells, where there was a smaller, but still highly significant overlap between bivalent domains and regions that have higher H3K27me3 in H3.3K27M cells (Supplementary Fig. 3d). Gene ontology analysis for genes linked to bivalent domains that had H3.3K27M-associated H3K27me3 showed a strong enrichment for genes involved in anterior/posterior pattern specification, primarily *HOX* family genes (see *HOXD1* as an example in Fig. 2e). Correlating with the decrease in the repressive modification H3K27me3 with reversion of K27M to WT, there were 10 *HOX* family gene transcripts that were significantly higher in Line XIII WT versus K27M cells and none that were significantly downregulated (Fig. 2f) versus isogenic parental K27M cells [10]. Three HOX transcripts had significantly higher expression in Line XVII WT compared to K27M (Supplemental Fig. 3e). Notably, while H3K27me3 levels are higher globally on chromatin in Line XIII WT cells compared to isogenic Line XIII K27M (full chromosome maps shown in Supplemental Fig. 4), the bivalent domains within exhibited selectively higher H3K27me3 in Line XIII K27M cells compared to WT (Supplemental Fig. 4 zoomed insets; bivalent domains in 5a, b).

While global cellular H3K4me3 levels were not changed between Line XIII H3.3K27M and WT cells (Supplemental Fig. 5c), we wanted to determine whether H3K4me3 chromatin occupancy changed specifically at bivalent domains with reversion of K27M to WT so we also performed H3K4me3 ChIP-seq in Line XIII WT cells and plotted the normalized H3K4me3 signal at Line XIII bivalent domains for Line XIII H3.3K27M and WT cells, as well as Line VI and Line XVII for comparison to these two other H3.3K27M cell lines. We found that the H3K4me3 signal was generally higher in Line XIII WT compared to the K27M cells (Supplemental Fig. 5d). We also found that the profile of H3K4me3 peaks specifically at *HOX* loci was very different in the Line XIII WT cells compared to XIII H3.3K27M and the other K27M cell lines VI and XVII. Fitting with this pattern, the XIII WT profiles were consistent with published H3K4me3 data from another H3.3WT DMG line (Line 48) (Supplemental Fig. 5e). The increase in H3K4me3 at bivalent domains, and the increase in H3K4me3 peaks within *HOX* loci in H3.3WT cells is consistent with the increase in expression of a number of *HOX* genes in WT cells compared to H3.3K27M cells (Fig. 2f and Supplemental Fig. 3e).

We next sought to understand potential mechanisms for the observed increase in H3K27me3 in DMG cells with H3.3K27M at a subset of the bivalent regions. We performed motif analysis on regions of open chromatin within bivalent domains, comparing those with increased H3K27me3 and those without a change in H3K27me3. Using MEME-ChIP, we found that bivalent regions without a change in H3K27me3 were enriched in a single motif with similarity to the MTF1 motif (Fig. 2g). In contrast, bivalent regions that have higher H3K27me3 in H3.3K27M cells were enriched in several other motifs, including notably the CTCF motif (Fig. 2h).

**Fig. 3:**
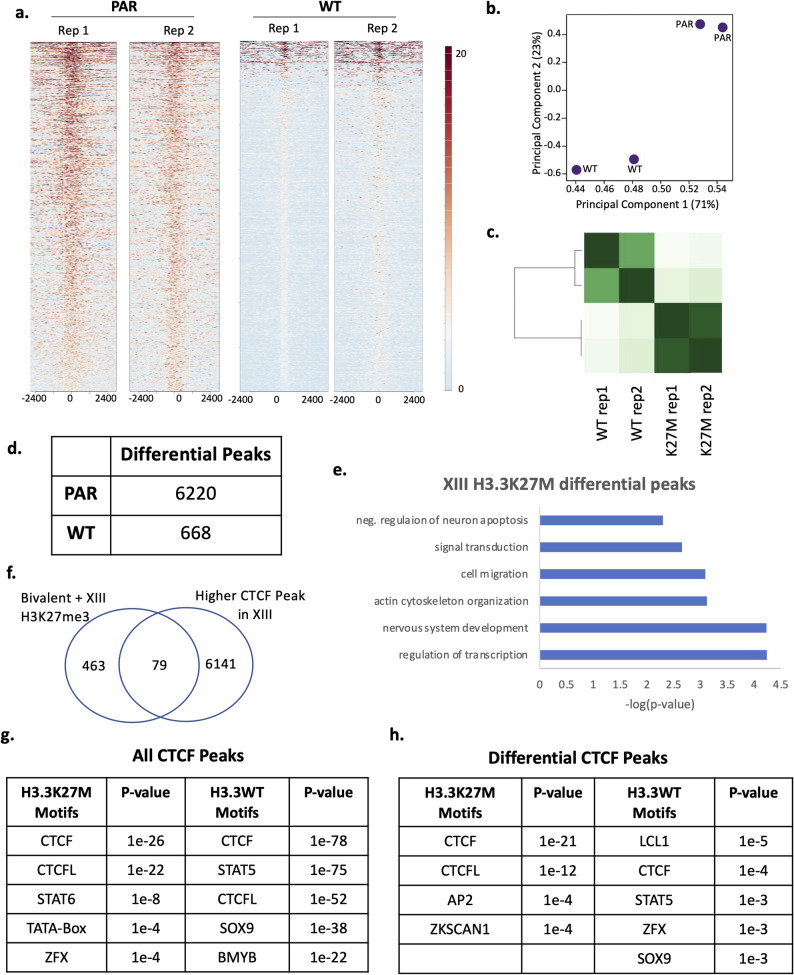
A subset of CTCF peaks are diff erentially enriched in Line XIII H3.3K27M cells compared toWT. **a** Density plots of CTCF signal. **b** PCA plot of CTCF CUT&RUN samples from Line XIII H3.3K27M andLine XIII WT cells. **c** Heatmap of CTCF CUT&RUN sample clustering. **d** Diff erential peaks identifi ed byDiff Bind for CTCF binding in Line XIII H3.3K27M and Line XIII WT cells. **e** Gene ontology analysis forgenes linked to peaks with significantly increased binding in H3.3K27M cells compared to WT. **f** Overlapof bivalent domains with increased H3K27me3 with upregulated CTCF peaks in XIII cells compared toXIII-WT. **g** Enriched motifs in all CTCF peaks in H3.3K27M and H3.3WT cells. **h** Enriched motifs in peakswith signifi cantly increased CTCF binding in H3.3K27M cells or H3.3WT cells.

### Evidence that K27M drives expansive ectopic CTCF binding in DMG

To begin to study potential connections between K27M and CTCF such as possible effects of H3.3K27M on localization of CTCF as well as the roles of CTCF at bivalent domains, we performed CUT&RUN with a CTCF antibody (antibody validation in Supplemental Fig. 6a) in isogenic Line XIII H3.3K27M and WT cells in duplicate. In the H3.3K27M cells, 11,970 and 12,715 CTCF peaks were identified in the two replicates, with 8557, or approximately 70% of CTCF peaks, overlapping between the two samples (Supplemental Fig. 6b). In Line XIII WT cells 15,434 and 18,226 CTCF peaks were identified in the two replicates and 7891 peaks overlapped between the two (Supplemental Fig. 6b). PCA analysis and hierarchical clustering of peaks show the H3.3K27M replicates clustered together and away from the H3.3WT replicates (Fig. 3a-c). We identified peaks with significantly different levels of CTCF binding between Line XIII H3.3K27M and H3.3WT cells using DiffBind [49]. In this analysis, 6220 peaks were identified with significantly higher CTCF binding in H3.3K27M cells compared to WT, but only 668 peaks had significantly higher CTCF in WT compared to K27M cells (Fig. 3d), despite no difference in *CTCF* expression between the cell types (Supplemental Fig. 6c).

Peaks with increased CTCF in H3.3K27M were found near genes enriched in functional categories related to nervous system development, transcriptional regulation, cell migration, and signal transduction (Fig. 3e). While there were no significantly enriched functional categories for peaks with increased CTCF in H3.3WT, likely due to the low number of such peaks, genes near these peaks were involved in processes like neuron differentiation and synaptic membrane transmission (Supplemental Fig. 6d).

We next overlapped the CTCF peaks with the bivalent domains in Line XIII cells. Of the 807 bivalent domains, 171 had CTCF binding (Supplemental Fig. 6e). In looking specifically at bivalent domains with increased H3K27me3 in Line XIII K27M compared to isogenic XIII-WT cells, 103 were CTCF-bound, and 79 of these were sites of significantly increased CTCF binding in XIII compared to XIII-WT cells (Supplemental Fig. 6f and 3f), suggesting a potential role for CTCF in regulating H3K27me3 levels at a subset of bivalent domains.

The most highly enriched motifs in CTCF peaks in both Line XIII H3.3K27M and WT were CTCF and CTCF binding factor like (CTCFL), providing further validation of our CTCF CUT&RUN data (Fig. 3g and Supplemental Table 4). CTCF peaks were also enriched in STAT family motifs in both cell types (Fig. 3g and Supplemental Table 4), while CTCF peaks in Line XIII H3.3WT cells were also enriched for SOX family motifs (Fig. 3g and Supplemental Table 4). We next looked at the subset of peaks showing differential CTCF binding in each cell type. Peaks with significantly higher CTCF binding in Line XIII K27M cells were enriched in motifs for AP2 and ZKSCAN1, in addition to CTCF and CTCFL (Fig. 3h and Supplemental Table 5). In contrast, peaks with significantly higher CTCF binding in Line XIII WT cells had significant enrichment of LCL1, ZFX, and SOX motifs (Fig. 3h and Supplemental Table 5).

### Impact of CTCF on chromatin domains in DMG

**Fig. 4:**
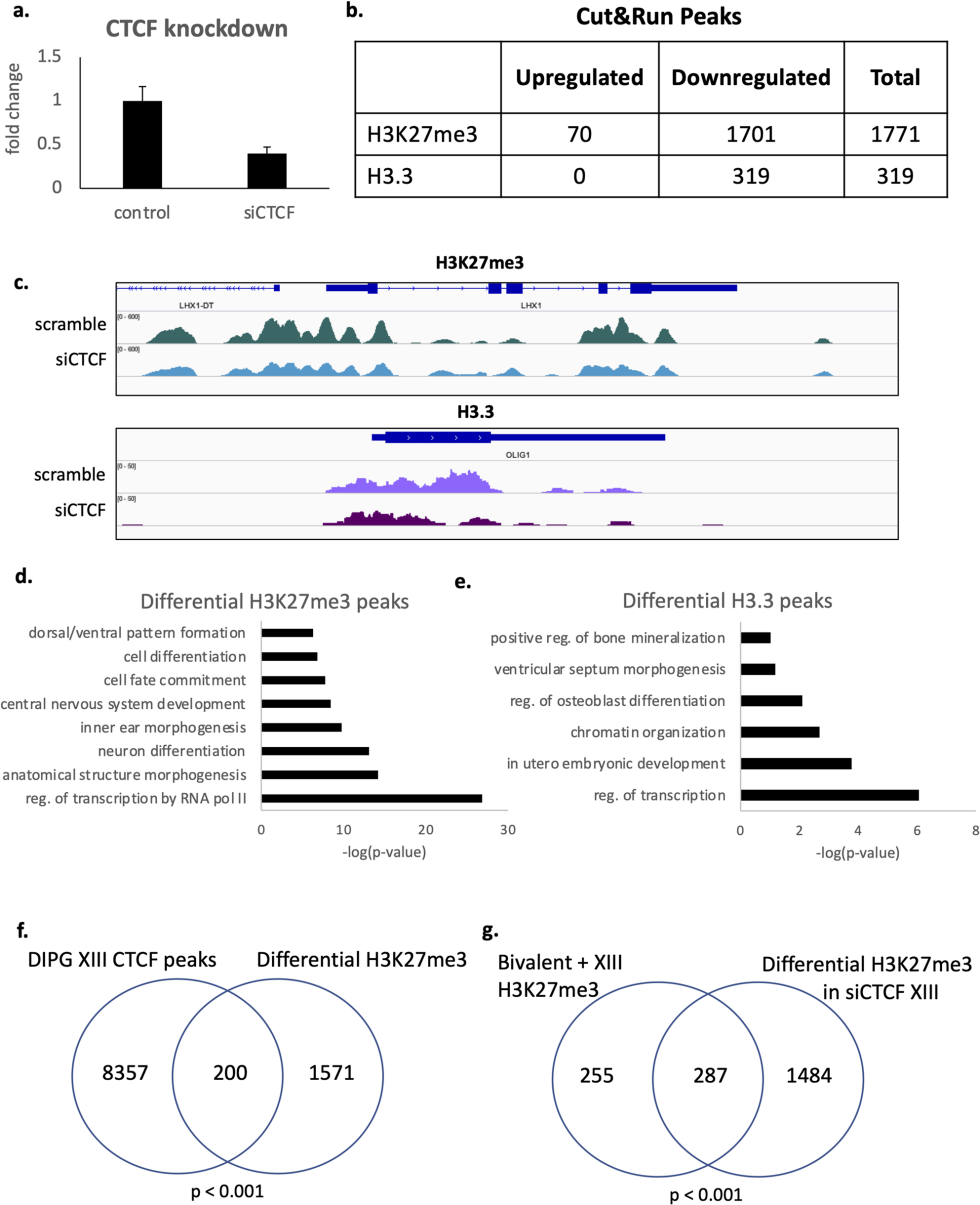
CTCF knockdown primarily causes reductions in H3.3 and H3K27me3 at a subset of regions. a. CTCF knockdown measured by qPCR in cells transfected with scramble control or CTCF siRNA, n=2. b. Regions with significantly increased or decreased H3.3 or H3K27me3 after CTCF knockdown compared to cells transfected with scramble control, as assessed by Cut&Run, n=2. c. Track image of sites with differential H3K27me3 or H3.3, one replicate shown for each cell type. d. Gene onology analysis for genes overlapping sites of differential H3K27me3 enrichment. e. Gene ontology analysis for genes overlapping sites of differential H3.3 enrichment. f. Overlap of CTCF peaks in DIPG XIII cells and regions with differential H3.3 enrichment upon CTCF knockdown. g. Overlap of CTCF peaks in DIPG XIII cells and regions with differential H3K27me3 enrichment upon CTCF knockdown.

To study the effects of CTCF on chromatin domains, we knocked down CTCF in parental K27M XIII DMG cells and performed CUT&RUN for H3.3 and H3K27me3 in duplicate (Fig. 4a, b and Supplemental Table 6). Approximately 50% knockdown of *CTCF* transcript and more modest drops in CTCF protein levels, likely due to the very long half-life of CTCF protein, were achieved (Fig. 4a and Supplemental Fig. 7a-b). Despite only moderate knockdown, using DiffBind, we identified 1771 peaks with differential H3K27me3 signal between control siRNA-transfected and CTCF knockdown cells, representing approximately 10% of total H3K27me3 peaks (Supplemental Table 6). Of the 1771 differential peaks, 1701 were depleted and 70 enriched upon CTCF knockdown (Fig. 4b). We also identified 319 regions with differential H3.3 signal upon CTCF knockdown; all were depleted with reduction in CTCF (Fig. 4b).

Only 24 regions showed both differential H3.3 and H3K27me3 in response to CTCF knockdown (Supplemental Fig. 7c). Examples of tracks with differential H3K27me3 or H3.3 upon CTCF knockdown are shown in Fig. 4c. Genes overlapping regions with differential H3K27me3 were enriched in categories related to cell and nervous system development, as well as transcriptional regulation (Fig. 4d). Genes overlapping regions with differential H3.3 were also enriched in GO categories related to development and transcriptional regulation; additionally, they were enriched in the chromatin organization category (Fig. 4e). Almost 50% of differential H3.3 sites were also sites of CTCF binding in XIII cells (Supplemental Fig. 7d), suggesting a strong connection between the two factors.

In contrast, only about 11% of differential H3K27me3 peaks overlapped with CTCF binding sites in XIII cells (Fig. 4f), but this was still a statistically significant overlap. We next looked at the effect of CTCF knockdown on bivalent regions with increased H3K27me3 in XIII K27M cells; strikingly, in more than half of these bivalent domains H3K27me3 levels were affected (mostly decreased) by CTCF knockdown (Fig. 4g), suggesting that CTCF binding may influence H3K27me3 levels at bivalent regions in DMG.

### Assessing CTCF knockdown impacts on DMG biology and the transcriptome

To investigate the potential biological effects of CTCF in DMG, we used siRNA to knock down CTCF expression in XIII cells (Supplemental Figs. 7a-b, 8a). We found that CTCF transcript reduction to less than 50% of starting levels resulted in no consistent changes in cell viability (Supplemental Fig. 8a-b) across a range of siRNA concentrations.

**Fig. 5:**
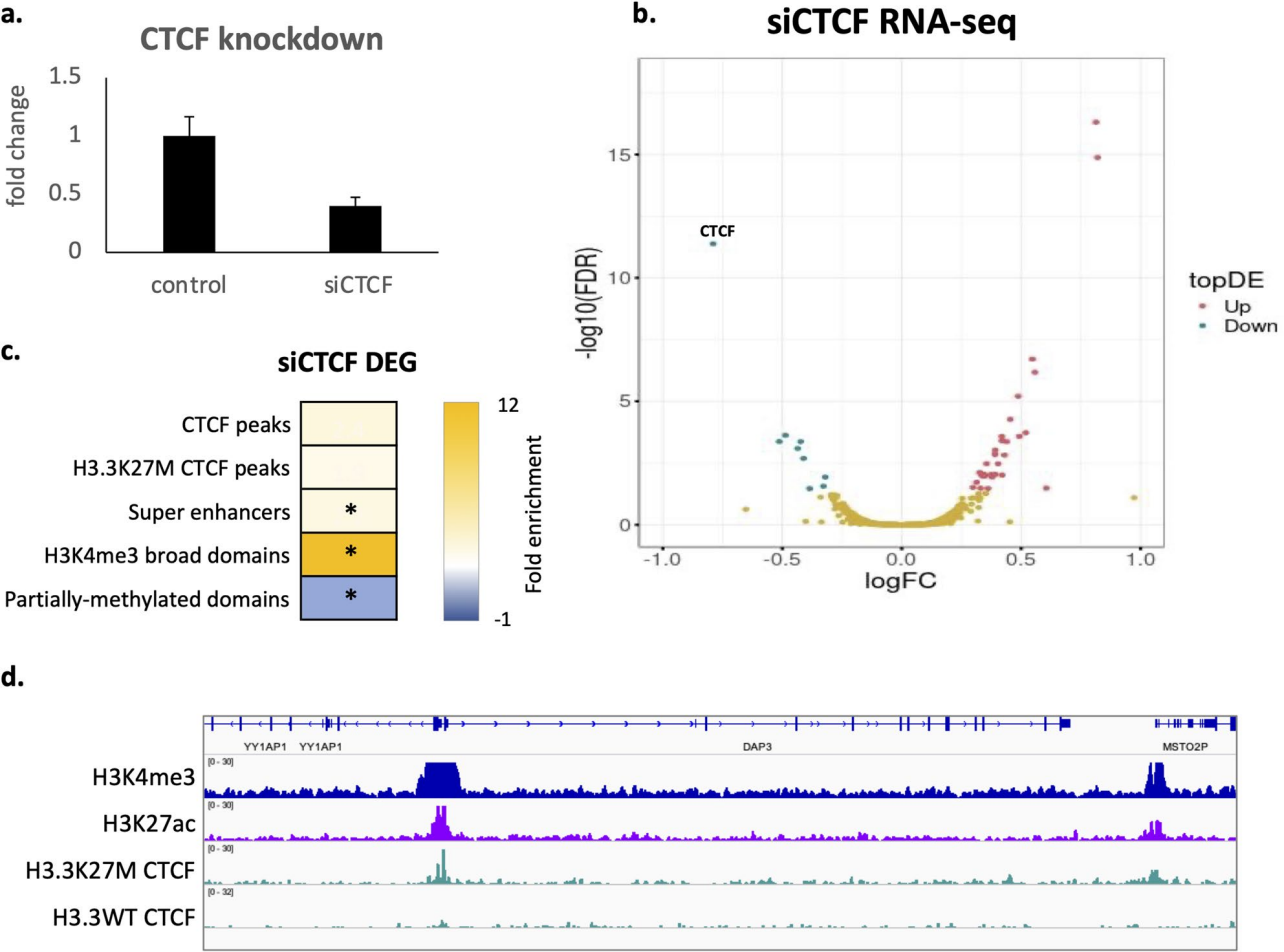
CTCF knockdown affects expression of genes linked to highly active regulatory domains. **a** Expression of CTCF in XIII K27M DMG cells transfected with either a scramble control or CTCF siRNA, *n* = 2. **b** Volcano plot highlighting differentially upregulated (red) and downregulated (blue) genes in RNA-seq data after CTCF knockdown. **c** Enrichment or significant under-enrichment of differentially expressed genes (DEG) across different chromatin domains. *p < .001. **d** Track image of H3K4me3, H3K27ac, and CTCF binding at DEG DAP3.

To define transcriptomic effects of CTCF knockdown and potential CTCF target genes in this context, we performed RNA-seq on line XIII K27M DMG cells with 50% CTCF loss-of-function via knock down (Fig. 5a). While control and CTCF loss-of-function DMG cells clearly segregated apart by PCA of the RNA-Seq results (Supplemental Fig. 8c), our analysis identified only 41 genes differentially expressed between scramble control and CTCF knockdown DMG samples (Table 1). Of these, 9 genes were downregulated and 32 upregulated, with CTCF being the most down-regulated gene (Fig. 5b, Supplemental Table 6), suggesting that short-term knockdown of CTCF to 50% of starting levels has only modest effects on DMG cell gene expression despite its stronger impacts on specific epigenetic marks throughout the genome. Despite the small number of affected genes, gene set enrichment analysis (GSEA) revealed enrichment of DEG in gene lists related to NOTCH signaling and MYC targets (Supplemental Fig. 8d), which is intriguing as Notch signaling has previously been shown to be affected by the H3.3K27M mutation in DMG.

**Table 1:** DEG after CTCF knockdown in DMG

Gene	GeneID	logFC	logCPM	*P* value	FDR
CTCF	ENSG00000102974	− 0.7896315	7.08767937	4.96E−15	4.19E−12
UNC13A	ENSG00000130477	− 0.5128583	7.63060321	2.39E−06	0.00043197
ZBTB18	ENSG00000179456	− 0.4873341	7.23315113	8.62E−07	0.00024277
KIF21A	ENSG00000139116	− 0.4354931	7.70164399	5.55E−06	0.00082815
WWP2	ENSG00000198373	− 0.423369	8.27163295	2.71E−06	0.00043468
PRKAB2	ENSG00000131791	− 0.411245	7.40669308	1.73E−05	0.00208387
ANKHD1	ENSG00000131503	− 0.3853267	7.62092527	0.00054567	0.0343999
AUTS2	ENSG00000158321	− 0.3280019	7.65093543	0.00039782	0.02801335
HERPUD1	ENSG00000051108	− 0.320238	8.19996518	0.00016012	0.01193845
CASC3	ENSG00000108349	0.2982521	7.63783624	0.00045792	0.03137347
NCOA5	ENSG00000124160	0.31358326	7.49752637	0.00026865	0.01945779
DAP3	ENSG00000132676	0.32625302	7.83997719	7.26E−05	0.00766775
ROBO1	ENSG00000169855	0.3301965	7.96642808	0.00050425	0.03363888
NT5DC2	ENSG00000168268	0.33334766	8.34915824	0.00011334	0.00990722
TNC	ENSG00000041982	0.33997356	10.6726963	0.00013587	0.01087417
QKI	ENSG00000112531	0.34487329	9.55648069	9.92E−05	0.00931706
PJA2	ENSG00000198961	0.34663006	7.69214316	0.00013727	0.01087417
STK35	ENSG00000125834	0.35058546	7.21948872	0.00089678	0.0541269
ZBED4	ENSG00000100426	0.35496947	7.60480659	3.08E−05	0.00342125
PTPN9	ENSG00000169410	0.36104705	7.35341201	0.00055637	0.0343999
SBNO1	ENSG00000139697	0.37322653	7.26610567	9.26E−05	0.00931706
EDIL3	ENSG00000164176	0.37355346	7.1609543	0.00012006	0.01014472
ZYX	ENSG00000159840	0.37820346	7.21790302	0.00015503	0.01190923
DNMT1	ENSG00000130816	0.39025573	8.54879661	1.08E−05	0.00143458
RTF1	ENSG00000137815	0.3914072	7.73199241	6.87E−06	0.00096782
SMAD2	ENSG00000175387	0.40331115	7.12892734	3.10E−05	0.00342125
SLC7A2	ENSG00000003989	0.41939598	7.51094336	1.16E−06	0.00026735
HIPK1	ENSG00000163349	0.41972495	7.48022114	1.96E−06	0.00039802
LSM14A	ENSG00000262860	0.42010026	7.20506188	0.00010846	0.00981962
CNOT3	ENSG00000088038	0.42234252	7.47692765	2.04E−06	0.00039802
SLC41A1	ENSG00000133065	0.4300355	7.16471771	1.21E−05	0.0015398
TNRC6A	ENSG00000090905	0.43960226	7.87538649	2.74E−06	0.00043468
TRIB2	ENSG00000071575	0.45426638	8.10522026	1.50E−07	5.44E−05
GNB1	ENSG00000078369	0.48717766	9.30042563	1.50E−08	6.33E−06
TEAD1	ENSG00000187079	0.49268065	7.32558741	1.07E−06	0.00026735
GLIS3	ENSG00000107249	0.51971195	7.2732939	6.00E−07	0.00019001
ALCAM	ENSG00000170017	0.54692828	8.33069974	3.11E−10	1.97E−07
NID1	ENSG00000116962	0.55771875	7.90938175	1.32E−09	6.70E−07
NDC1	ENSG00000058804	0.60529807	7.21884403	0.0005177	0.03365048
YWHAH	ENSG00000128245	0.81346037	7.6144235	1.93E−20	4.89E−17
TLN1	ENSG00000137076	0.81981446	8.93861396	1.05E−18	1.33E−15

We next overlapped the differentially expressed genes (DEG) found in CTCF knockdown DMG cells with CTCF CUT&RUN peaks, as well as chromatin domains like super enhancers, H3K4me3 broad domains, and partially methylated domains (PMDs). There was a significant enrichment of super enhancers and H3K4me3 broad domains at DEG with reduction of CTCF, while there was a depletion of PMDs at DEG (Fig. 5c). Several DEG are in regions identified as both super enhancers and H3K4me3 broad domains, which likely represent regions of very high activity and regulation (Figs. 5c-d). It may be that even subtle changes in CTCF-influenced gene expression at these highly regulated domains have an outsized effect on cell dynamics including cancer-related functions.

### H3.3 and CTCF show strong correlation and are found in proximity across the genome in DMG cells

**Fig. 6:**
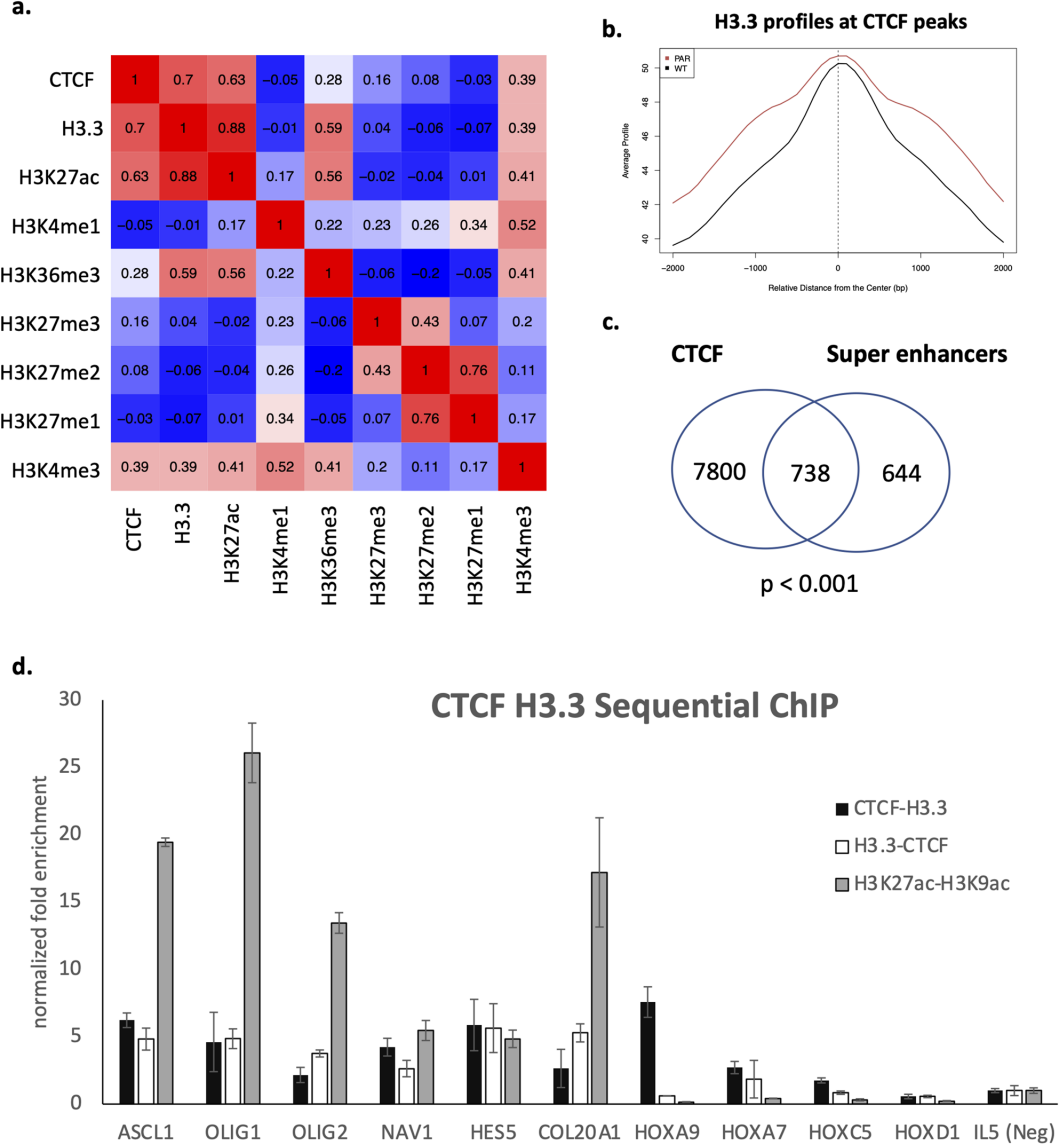
H3.3 and CTCF show strong genomic correlation and are found in close proximity at many genes in Line 13 DIPG cells. **a** Heatmap plotting genome-wide correlation coefficient for each histone modification, histone H3.3, and CTCF in Line 13 H3.3K27M cells. **b** Line 13 PAR and 13 WT H3.3 ChIP-seq signal profiles at CTCF peaks. **c** Overlap of CTCF peaks and super enhancers in Line 13 H3.3K27M cells. **d** Normalized fold enrichment of sequential ChIP with CTCF and H3.3 at predicted co-bound sites. All values normalized to negative control sequential IgG-IgG pull down. Sequential pull down of H3K27ac-H3K9ac as positive control.

To investigate the relationship between CTCF and other chromatin modifications in Line XIII H3.3K27M cells, we compiled a set of published epigenetic datasets in Line XIII H3.3K27M cells to calculate the correlation coefficient values for each combination of two datasets across the genome using Cistrome (Fig. 6a). We found a very strong genome-wide correlation between CTCF, H3.3, and H3K27ac. H3.3 is found at CTCF binding sites in both Line XIII H3.3K27M and isogenic H3.3WT cells (Fig. 6b).

Due to the strong overlap of CTCF with the histone modification H3K27ac, which is found at super enhancers and other active regulator regions, we overlapped our CTCF peaks with previously-identified super enhancers in Line XIII H3.3K27M cells [37] and found that more than half of the super enhancers contain one or more CTCF peaks, which is significantly higher than would be expected by chance (Fig. 6c). We also identified a strong overlap of CTCF binding with H3K4me3 broad domains (Supplemental Fig. 9a).

To assess potential co-binding of CTCF and H3.3 on chromatin, we performed sequential ChIP in XIII cells for the two factors at predicted co-binding sites including *HOX* loci based on our CTCF CUT&RUN and H3.3 ChIP-seq data and normalized the results to an IgG sequential ChIP as a negative control. Strikingly, CTCF and H3.3 were co-enriched at the majority of sites tested, and in most cases, the enrichment was similar regardless of which factor was immunoprecipitated first (Fig. 6f and Supplemental Fig. 9c). As a positive control, we also see strong enrichment at these same locations for sequential ChIP with H3K27ac and H3K9ac antibodies (Fig. 6d and Supplemental Fig. 9c). To determine whether CTCF and H3.3 associate with each other on chromatin in other cells, we repeated the sequential ChIP experiments in Line XVII cells, finding co-enrichment of CTCF and H3.3 at a number of the sites identified in Line XIII cells (Supplemental Fig. 9d, e).

### ChromHMM identifies potential boundary domain between active and repressed chromatin

To obtain a more comprehensive view of the combinatorial patterns of epigenetic marks in DMG cells and potential relationships with H3.3, we expanded our analysis to include a large set of epigenetic data in Line XIII cells, This analysis included the CTCF CUT&RUN data and H3K4me3 ChIP-seq data, our previously published ATAC-seq, H3.3 and H3K27me3 ChIP-seq data, and publicly available datasets for DNA methylation and other chromatin modifications all in Line XIII cells. We then used ChromHMM to identify and annotate chromatin domains in DMG cells. ChromHMM uses a hidden Markov model to learn chromatin states in a cell type based on the combinations of epigenetic modifications as well as their spatial context and has been used extensively to catalog cell states across ENCODE cell lines [12].

**Fig. 7:**
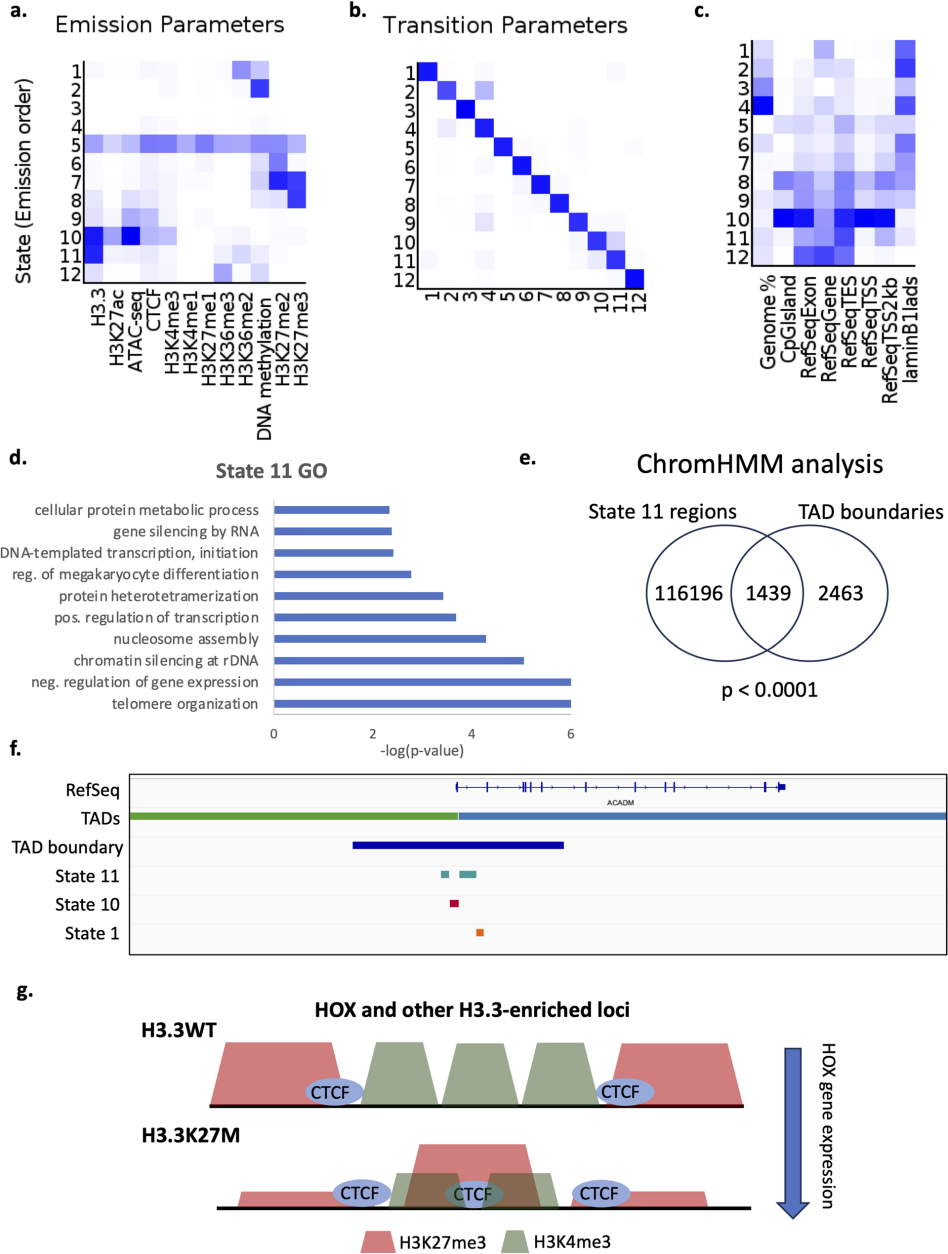
ChromHMM identifies potential boundary domain between active and repressed chromatin that is highly enriched in H3.3 and CTCF in DIPG Line 13 cells. **a** Heatmap showing emission parameters for each of the 12 identified states and their associated chromatin modifications. Dark blue is indicative of a higher probability that a modification is found in a given state. **b** Transition parameters showing the probability of transitioning from a given state to each other state. Dark indicates higher probability. **c** Heatmap showing enrichment of each state in various genomic features. **d** Gene ontology analysis for genes overlapping state 11 regions. **e** Overlap of State 11 regions and Topologically associated domain (TAD) boundaries **f** Track view of State 11 regions at TAD boundaries. **g** Model figure for H3.3-associated CTCF, H3K4me3, and H3K27me3 localization at bivalent domains.

Using all available datasets for DMG Line XIII, ChromHMM defined 12 unique chromatin states (Fig. 7a-c). Among these were several states associated with various combinations of repressive chromatin marks including H3K27me3 and DNA methylation (states 1,2, 6–8), and several states associated with active chromatin modifications like H3K4me3, H3K27ac, and ATAC-seq peaks (states 9 and 10) (Fig. 7a). As expected, regions associated with repressive marks were enriched in LAMINB1 domains found at the periphery of the nucleus, while regions associated with active marks were enriched in CpG islands, genes, and gene promoters (Fig. 7c).

States 10 and 11 were most strongly enriched for H3.3. Notably, our analysis also identified state 11 as having a unique combination of active and repressed marks, as well as CTCF binding and very strong enrichment of the H3.3 histone variant (Fig. 7a-c and Supplemental Fig. 9f, g). This state was strongly enriched both within gene bodies and within LAMINB1 associated domains, but not at transcription start sites. State 11 showed an increased probability of transition to states 9 and 10, marked by active chromatin marks (Fig. 7b), and also an increased probability of transition to state 1, marked by repressive DNA methylation and H3K36me2, suggesting state 11 may function as a boundary domain between active and repressed regions. State 11 regions were located in proximity to genes involved in functions like nucleosome assembly, regulation of transcription, and telomere organization (Fig. 7d). These categories are notable, given the striking enrichment of histone variant H3.3 in state 11 regions, the known role of H3.3 in the gene functions enriched in this state, and the potential roles for these gene functions in H3.3 mutant DMG.

To determine whether State 11 regions are enriched in known boundary domains, we identified topologically associated domains (TADs) as well as TAD boundaries from published HiC data in Line XIII cells [54]. When we overlapped these TAD boundaries with our State 11 regions, we identified a highly significant overlap between the two, adding further weight to the idea that State 11 regions serve as boundary domains within chromatin (Fig. 7e, f).

## Discussion

Our results identify key chromatin regulatory domains in DMG, including the characterization of H3K4me3 broad domains and roles for CTCF linked to H3.3 for the first time in DMG cells. The finding that H3K4me3 broad domains and super enhancers are primarily separate regulatory domains with distinct enriched motifs and putative gene targets suggests multiple gene regulatory networks are operating simultaneously in DMG cells, which are likely important considerations in designing treatment regimens. Super enhancer-mediated regulation is linked to genes involved in cell adhesion and cell migration functions; these are genes likely to be involved in the invasiveness of DMG cells and represent important potential pathways to target. H3K4me3 broad domains are linked to genes involved in chromatin remodeling and nucleosome assembly, and misregulation of these genes in tumorigenesis may contribute to the genomic instability observed in DMG cells with the H3.3K27M mutation. These results suggest that any epigenetics-based pharmacological approach targeting active regions would be more effective combining drugs that target H3K4me3 and H3K27ac. In addition, some past work potentially tied CTCF to H3K4me3 in K27M glioma, finding that H3K4me3 peaks unique to K27M cells were enriched for CTCF motifs [15].

We also found that bivalent domains in DMG cells, including within the *HOX* gene clusters, are repressed by the H3.3K27M mutation, and that this effect may be mediated by CTCF binding and H3K27me3. It was striking that so many bivalent domains in XIII cells were associated with increased H3K27me3 in the presence of H3.3K27M, as the H3.3K27M mutation has been associated with reduced levels of H3K27me3 globally in many studies and in our work here. There has been a long-standing puzzle in the field as to what focal increases in H3K27me3 might mean in DMG and how they are regulated. Our results suggest a role for CTCF related to focal H3K27me3 increases and associated gene repression with H3K27M as well as potential functional consequences for DMG cell biology (model in Fig. 7g).

Many genes have a bivalent state in stem cells and lose the state as the cells differentiate; it may be that the bivalent regions identified in our study take on an increased or altered form of bivalency, including elevated H3K27me3 in pre-cancerous stem or precursor-like cells bearing the H3.3K27M mutation as the cells maintain or acquire a less differentiated state during tumorigenesis, in spite of the global reductions in H3K27me3. This possibility of cells with H3.3K27M maintaining or taking on a more stem-like state is also consistent with the observed reduction in *HOX* gene expression and H3K4me3 levels at *HOX* loci in H3.3K27M K27M cells, as stem cells typically express lower levels of *HOX* genes than more differentiated cells. Loci that are bivalent but with relatively high H3K27me3 levels may represent a novel subclass of bivalent domains with unique functional status that are unlikely to be activated as long as the K27M mutation is present.

Our work implicating bivalent genes in DMG is consistent with previous work also pointing toward bivalency as a key mechanism in these glioma [31, 51]. Importantly, those studies further linked bivalent genes to inhibited differentiation of DMG, and targeted K27M loss of function allowed for some differentiation of DMG at least in part through changes in bivalent genes. Our studies further suggest that *HOX* genes may play a role in this dynamic, consistent with the idea that specific homeodomain proteins can contribute to oncogenesis in the brain including specifically in DMG [42].

A role for CTCF in regulating the structure of the *HOX* loci, as well as providing insulator function to control levels of H3K4me3 and H3K27me3 in other cell types have been previously described [39, 53]. Indeed, a previous study showed that CTCF can open chromatin in part by binding chromatin and incorporating H3.3 to replace histones with H3K27me3, thereby removing this repressive mark [55]. In our work, modest CTCF knockdown in DMG cells had overall mild effects and was primarily associated with a reduction in H3K27me3 at specific regions, with limited effects on gene expression. This suggests that CTCF is functionally in excess or there are robust compensatory mechanisms when its level is moderately reduced such as via CTCFL. It is also possible that CTCF regulatory mechanisms may be context dependent and that CTCF may act differently at distinct chromatin domains, particularly in cancers. There may also be differences with how CTCF interacts with the mutant H3.3 protein specifically in the DMG context. Such dynamics may also explain why, despite the strong evidence here for CTCF activities and coordinate H3.3 and CTCF functions in DMG downstream of K27M, we found that an approximately 50% reduction in CTCF levels by siRNA did not clearly impact DMG cell viability.

Our work highlights an interesting connection between CTCF and H3.3 in DMG. In our data, both CTCF and H3.3 are found at the edges of many gene clusters. We also find that mutant H3.3K27M affects both CTCF binding and H3K27me3 levels at bivalent domains including at the *HOX* gene clusters. This finding is consistent with studies showing CTCF stabilizes the Polycomb repressive complex 2 (PRC2) and H3K27me3 levels at *HOX* loci in other cell types [58].

Our work showing a strong association of H3.3 and CTCF genome-wide, as well as a direct interaction between H3.3 chaperone HIRA and co-binding of H3.3 and CTCF on chromatin suggest that the two factors act together to regulate chromatin structure and gene regulation, especially at gene clusters including the *HOX* loci. Future studies looking at the effects of deletion of CTCF binding sites on nearby H3.3 and H3K27me3 levels would provide additional mechanistic insight into the co-regulatory functions of CTCF and H3.3. Indeed, this association between H3.3 and CTCF, a factor with well characterized roles in chromatin structure, looping, and insulation at boundary regions, may help explain the observed effect of H3.3K27M mutation on genomic stability. The interaction between H3.3 and CTCF could also impact cell fate during different stages of DMG tumorigenesis.

ChromHMM has been used to identify and annotate chromatin domains across a wide range of cell types using a standardized set of input data including the most common and well-characterized histone modifications, DNase hypersensitivity, and DNA methylation. Since these initial studies, numerous additional histone modifications with unique functions and spatial patterns have been characterized and have the potential to further refine models of chromatin domains across the genome. Indeed, by incorporating the histone variant H3.3 and the histone modification H3K36me2, we have identified a unique boundary-type chromatin state that includes the combination of these two marks, as well as the presence of several more common modifications including DNA methylation and low levels of H3K4me3, H3K27ac, and CTCF. This boundary association of histone H3.3 is consistent with our findings of H3.3 localization at the edges of gene clusters and suggests that histone variants are also an important component of chromatin segmentation and domain separation. Part of the observed genomic instability in cells with mutant H3.3 may be due to perturbation of this function in chromatin domain organization and insulation.

Together, our results describe important aspects and functions of the complex chromatin landscape in DMG cells and document the effects of the H3.3K27M mutation on these chromatin domains. Key findings include identification of regulatory differences between super enhancers and H3K4me3 broad domains, finding that CTCF and H3.3 co-bind many targets, and that mutant H3.3 is associated with altered H3K27me3 at bivalent domains and gene expression changes, among other effects. Our findings suggest that in H3.3 mutant DMG, therapeutic strategies based on combinatorial drug treatments targeting multiple epigenetic enzymes and pathways are likely to be more effective than individual epigenetic drugs, due to the diverse effects of the H3.3K27M mutation on chromatin.

## Supplementary Information

Below is the link to the electronic supplementary material.

Supplementary Material 1

Supplementary Material 2

Supplementary Material 3

Supplementary Material 4

Supplementary Material 5

Supplementary Material 6

Supplementary Material 7

## Data Availability

All material, with appropriate MTA, will be made available upon request. All genomics data has already been deposited in GEO so is available: GSE285924, GSE285840, GSE285841, GSE326768.
