# Supplementary material for: Evidence for coordinate CTCF and histone H3.3 activities in K27M diffuse midline gliomas
Source: Acta Neuropathol Commun. 2026 May 2;14:132. doi: 10.1186/s40478-026-02290-2 (PMC13288838; doi:10.1186/s40478-026-02290-2)
Supplement: Supplementary file 1 — Supplementary Material 1 [file 40478_2026_2290_MOESM1_ESM.pptx]

## Slide 1
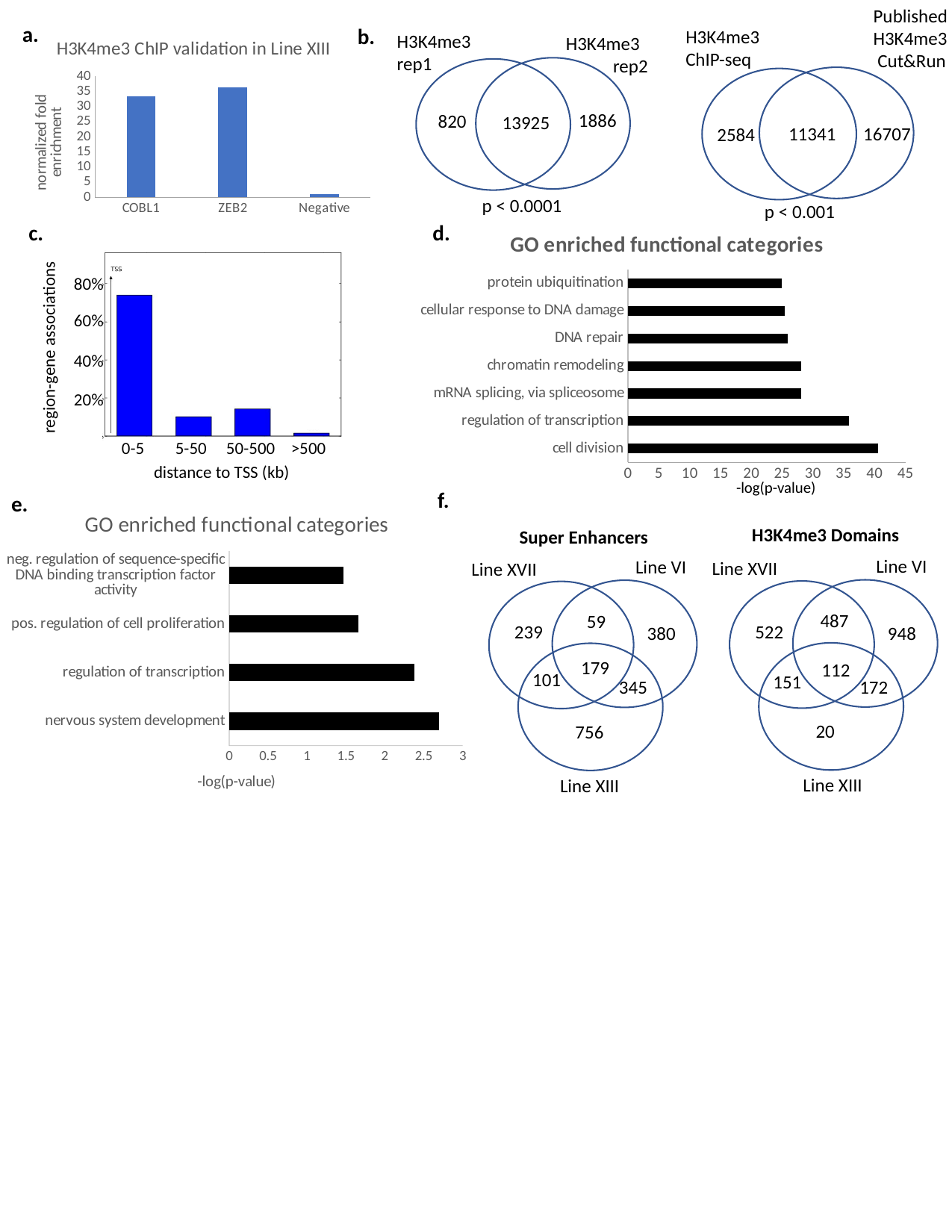

Published
H3K4me3
 Cut&Run
a.
b.
H3K4me3
ChIP-seq
H3K4me3
rep1
### Chart: H3K4me3 ChIP validation in Line XIII
| Category | |
|---|---|
| COBL1 | 33.47471821597397 |
| ZEB2 | 36.37814312810062 |
| Negative | 1.0 |H3K4me3
 rep2
1886
820
13925
11341
16707
2584
p < 0.0001
p < 0.001
c.
d.
### Chart: GO enriched functional categories
| Category | |
|---|---|
| cell division | 40.56863623584101 |
| regulation of transcription | 35.85387196432176 |
| mRNA splicing, via spliceosome | 28.065501548756433 |
| chromatin remodeling | 28.065501548756433 |
| DNA repair | 25.958607314841775 |
| cellular response to DNA damage | 25.42021640338319 |
| protein ubiquitination | 24.958607314841775 |
80%
60%
region-gene associations
40%
20%
0-5
5-50
50-500
>500
distance to TSS (kb)
-log(p-value)
f.
e.
### Chart: GO enriched functional categories
| Category | |
|---|---|
| nervous system development | 2.6989700043360187 |
| regulation of transcription | 2.3767507096020997 |
| pos. regulation of cell proliferation | 1.6575773191777938 |
| neg. regulation of sequence-specific DNA binding transcription factor activity | 1.4685210829577449 |H3K4me3 Domains
Super Enhancers
Line VI
Line VI
Line XVII
Line XVII
487
59
522
239
948
380
179
112
101
151
172
345
20
756
Line XIII
Line XIII

## Slide 2
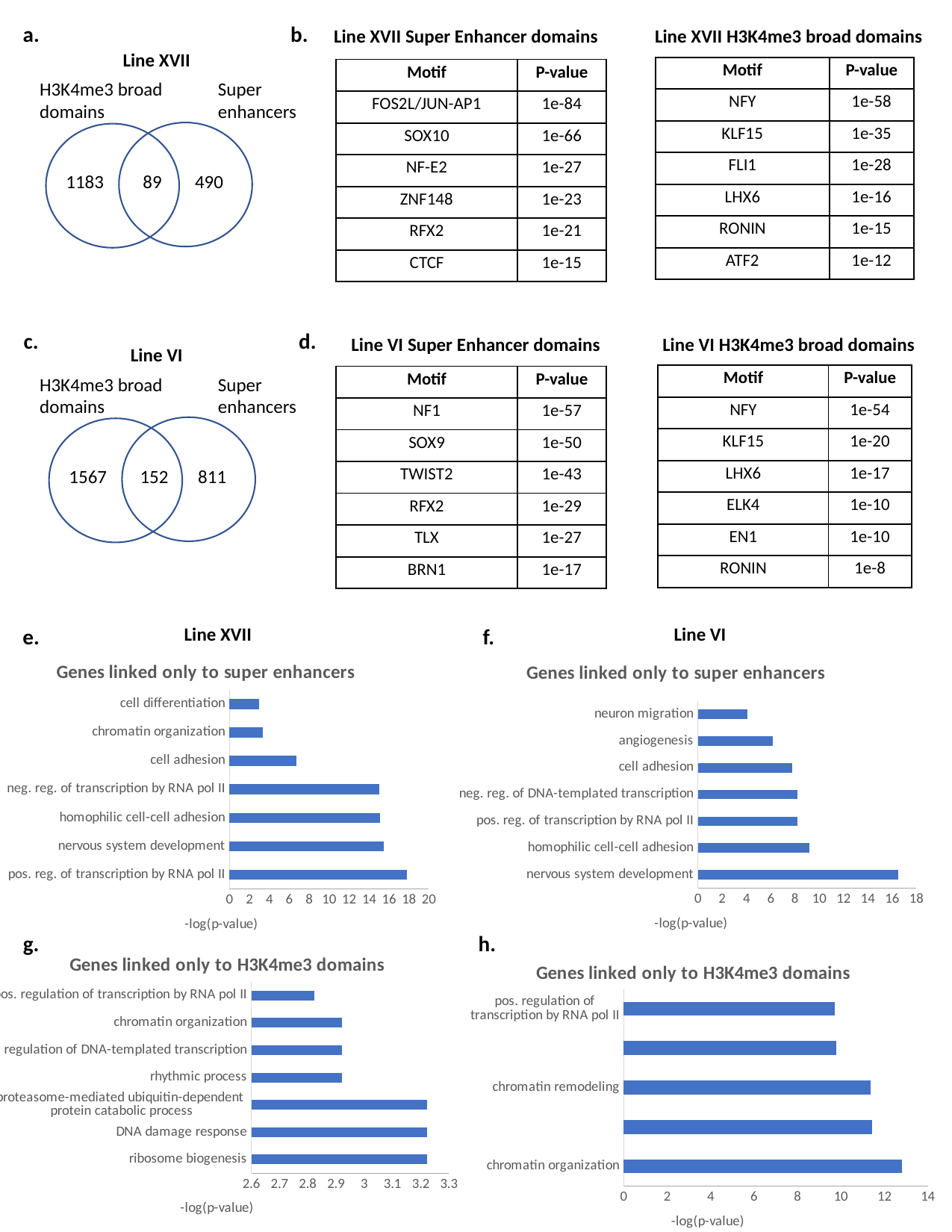

a.
b.
Line XVII Super Enhancer domains
Line XVII H3K4me3 broad domains
Line XVII
| Motif | P-value |
| --- | --- |
| NFY | 1e-58 |
| KLF15 | 1e-35 |
| FLI1 | 1e-28 |
| LHX6 | 1e-16 |
| RONIN | 1e-15 |
| ATF2 | 1e-12 |
| Motif | P-value |
| --- | --- |
| FOS2L/JUN-AP1 | 1e-84 |
| SOX10 | 1e-66 |
| NF-E2 | 1e-27 |
| ZNF148 | 1e-23 |
| RFX2 | 1e-21 |
| CTCF | 1e-15 |
H3K4me3 broad
domains
Super
enhancers
1183
89
490
c.
d.
Line VI Super Enhancer domains
Line VI H3K4me3 broad domains
Line VI
| Motif | P-value |
| --- | --- |
| NFY | 1e-54 |
| KLF15 | 1e-20 |
| LHX6 | 1e-17 |
| ELK4 | 1e-10 |
| EN1 | 1e-10 |
| RONIN | 1e-8 |
| Motif | P-value |
| --- | --- |
| NF1 | 1e-57 |
| SOX9 | 1e-50 |
| TWIST2 | 1e-43 |
| RFX2 | 1e-29 |
| TLX | 1e-27 |
| BRN1 | 1e-17 |
H3K4me3 broad
domains
Super
enhancers
1567
152
811
Line XVII
Line VI
e.
f.
### Chart: Genes linked only to super enhancers
| Category | |
|---|---|
| pos. reg. of transcription by RNA pol II | 17.82390874094432 |
| nervous system development | 15.468521082957745 |
| homophilic cell-cell adhesion | 15.09151498112135 |
| neg. reg. of transcription by RNA pol II | 15.0 |
| cell adhesion | 6.721246399047171 |
| chromatin organization | 3.3565473235138126 |
| cell differentiation | 3.0 |
### Chart: Genes linked only to super enhancers
| Category | |
|---|---|
| nervous system development | 16.494850021680094 |
| homophilic cell-cell adhesion | 9.161150909262744 |
| pos. reg. of transcription by RNA pol II | 8.187086643357144 |
| neg. reg. of DNA-templated transcription | 8.187086643357144 |
| cell adhesion | 7.795880017344075 |
| angiogenesis | 6.187086643357144 |
| neuron migration | 4.086186147616283 |g.
h.
### Chart: Genes linked only to H3K4me3 domains
| Category | |
|---|---|
| chromatin organization | 12.823908740944319 |
| DNA damage response | 11.42021640338319 |
| chromatin remodeling | 11.3767507096021 |
| neg. regulation of transcription by RNA pol II | 9.769551078621726 |
| pos. regulation of transcription by RNA pol II | 9.698970004336019 |
### Chart: Genes linked only to H3K4me3 domains
| Category | |
|---|---|
| ribosome biogenesis | 3.2218487496163566 |
| DNA damage response | 3.2218487496163566 |
| proteasome-mediated ubiquitin-dependent
protein catabolic process | 3.2218487496163566 |
| rhythmic process | 2.9208187539523753 |
| neg. regulation of DNA-templated transcription | 2.9208187539523753 |
| chromatin organization | 2.9208187539523753 |
| pos. regulation of transcription by RNA pol II | 2.8239087409443187 |

## Slide 3
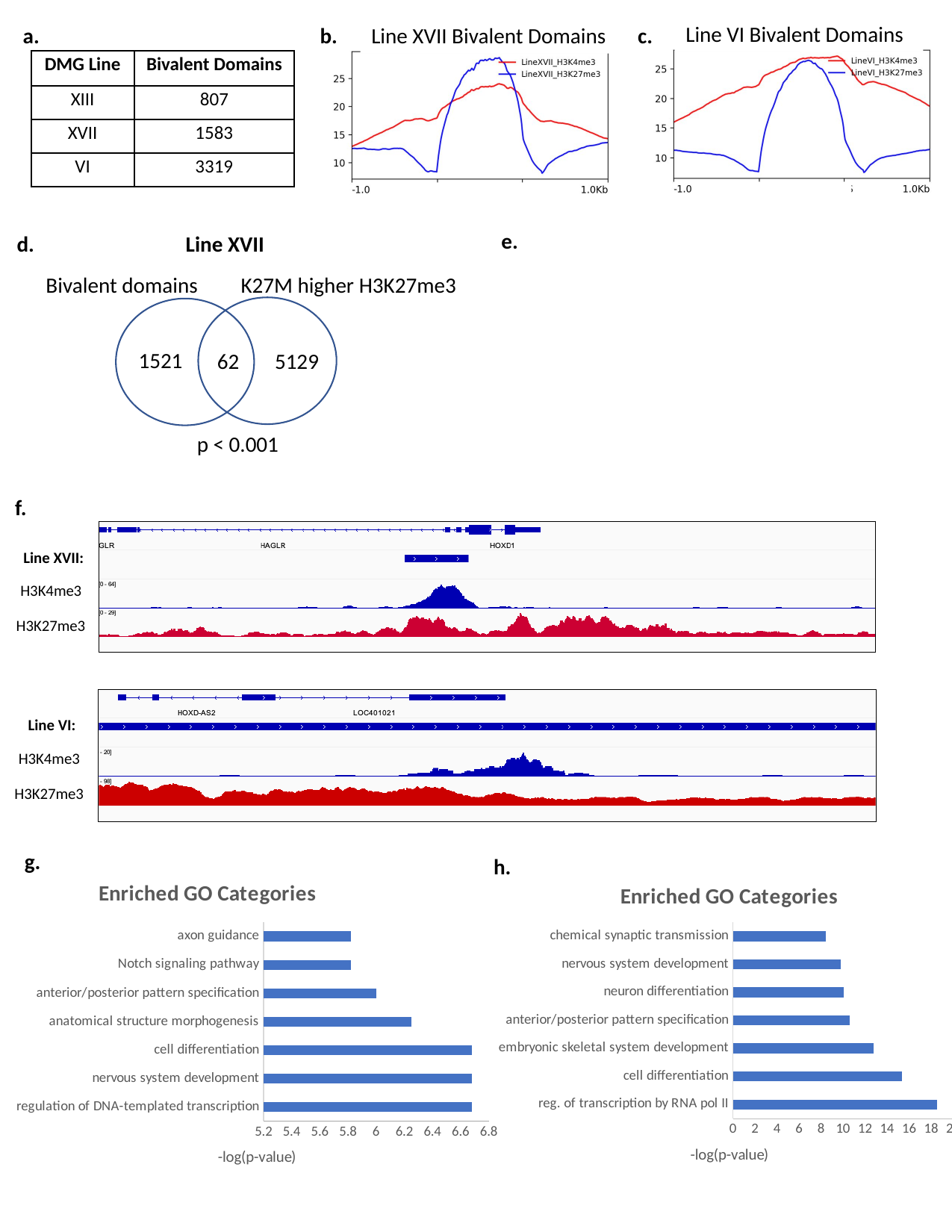

Line VI Bivalent Domains
Line XVII Bivalent Domains
a.
b.
c.
| DMG Line | Bivalent Domains |
| --- | --- |
| XIII | 807 |
| XVII | 1583 |
| VI | 3319 |
e.
Line XVII
d.
 Bivalent domains
K27M higher H3K27me3
1521
62
5129
p < 0.001
f.
Line XVII:
H3K4me3
H3K27me3
Line VI:
H3K4me3
H3K27me3
g.
h.
### Chart: Enriched GO Categories
| Category | |
|---|---|
| regulation of DNA-templated transcription | 6.6777807052660805 |
| nervous system development | 6.6777807052660805 |
| cell differentiation | 6.6777807052660805 |
| anatomical structure morphogenesis | 6.251811972993799 |
| anterior/posterior pattern specification | 6.0 |
| Notch signaling pathway | 5.823908740944319 |
| axon guidance | 5.823908740944319 |
### Chart: Enriched GO Categories
| Category | |
|---|---|
| reg. of transcription by RNA pol II | 18.55284196865778 |
| cell differentiation | 15.3767507096021 |
| embryonic skeletal system development | 12.769551078621726 |
| anterior/posterior pattern specification | 10.585026652029182 |
| neuron differentiation | 10.045757490560675 |
| nervous system development | 9.823908740944319 |
| chemical synaptic transmission | 8.443697499232712 |

## Slide 4
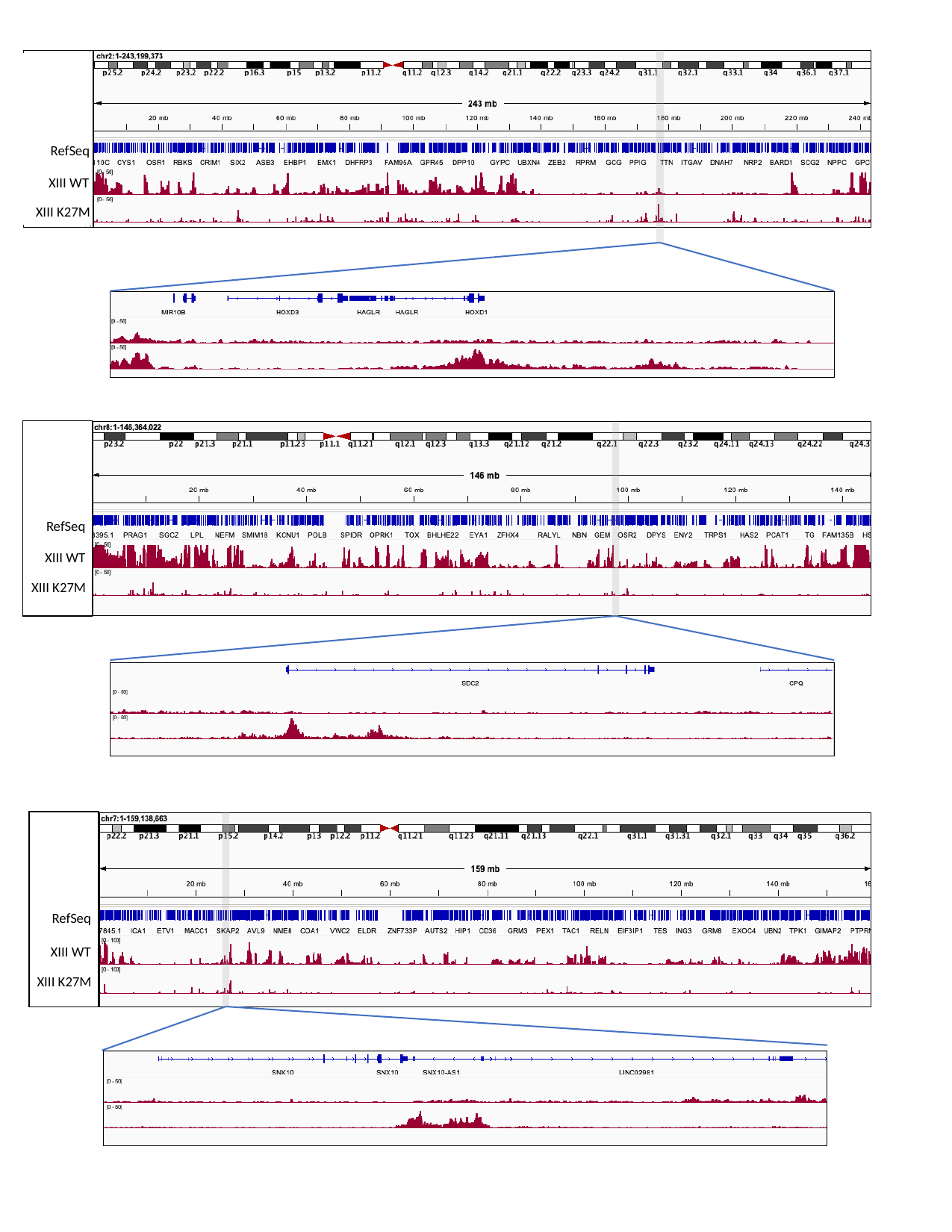

RefSeq
XIII WT
XIII K27M
RefSeq
XIII WT
XIII K27M
RefSeq
XIII WT
XIII K27M

## Slide 5
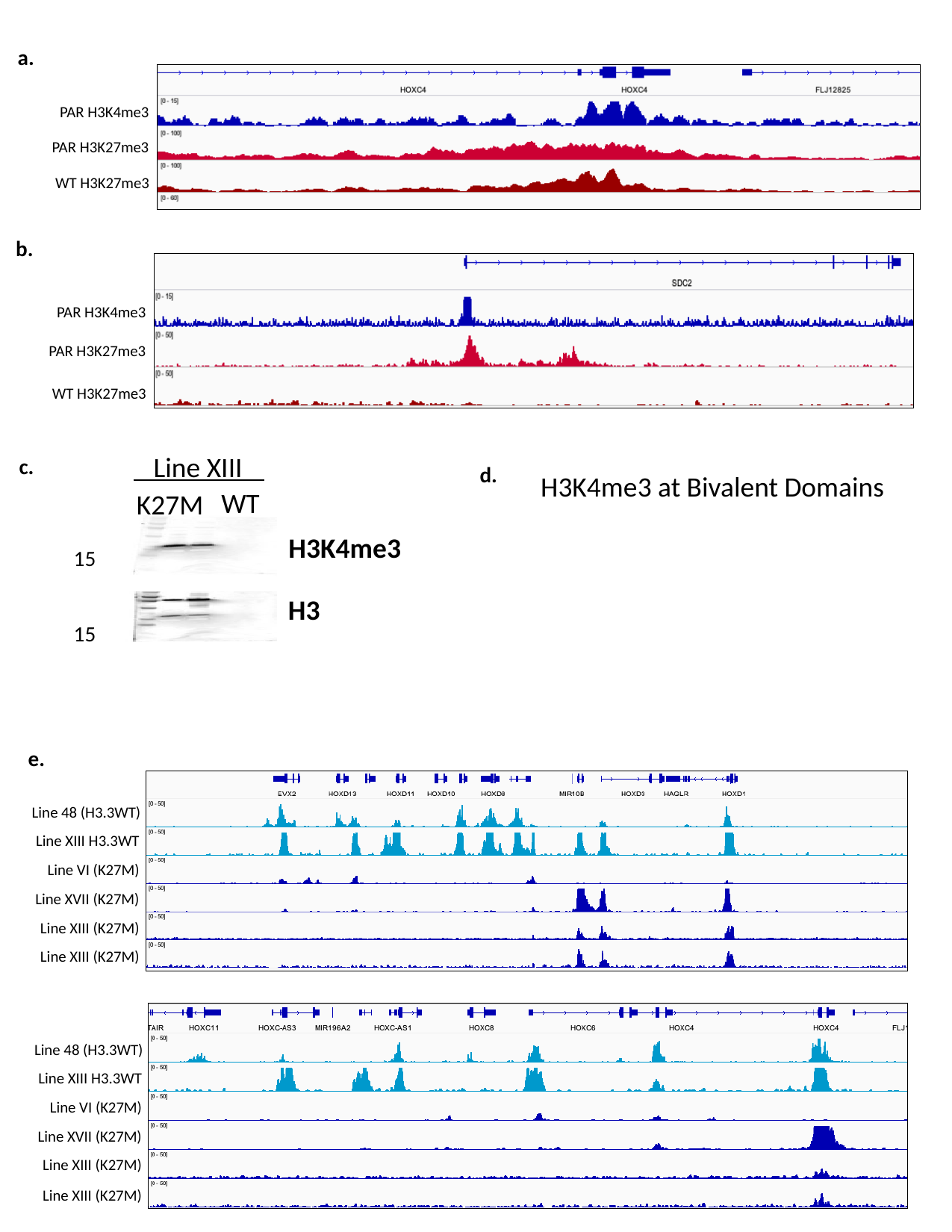

a.
PAR H3K4me3
PAR H3K27me3
WT H3K27me3
b.
PAR H3K4me3
PAR H3K27me3
WT H3K27me3
Line XIII
c.
d.
H3K4me3 at Bivalent Domains
WT
K27M
H3K4me3
15
H3
15
e.
Line 48 (H3.3WT)
Line XIII H3.3WT
Line VI (K27M)
Line XVII (K27M)
Line XIII (K27M)
Line XIII (K27M)
Line 48 (H3.3WT)
Line XIII H3.3WT
Line VI (K27M)
Line XVII (K27M)
Line XIII (K27M)
Line XIII (K27M)

## Slide 6
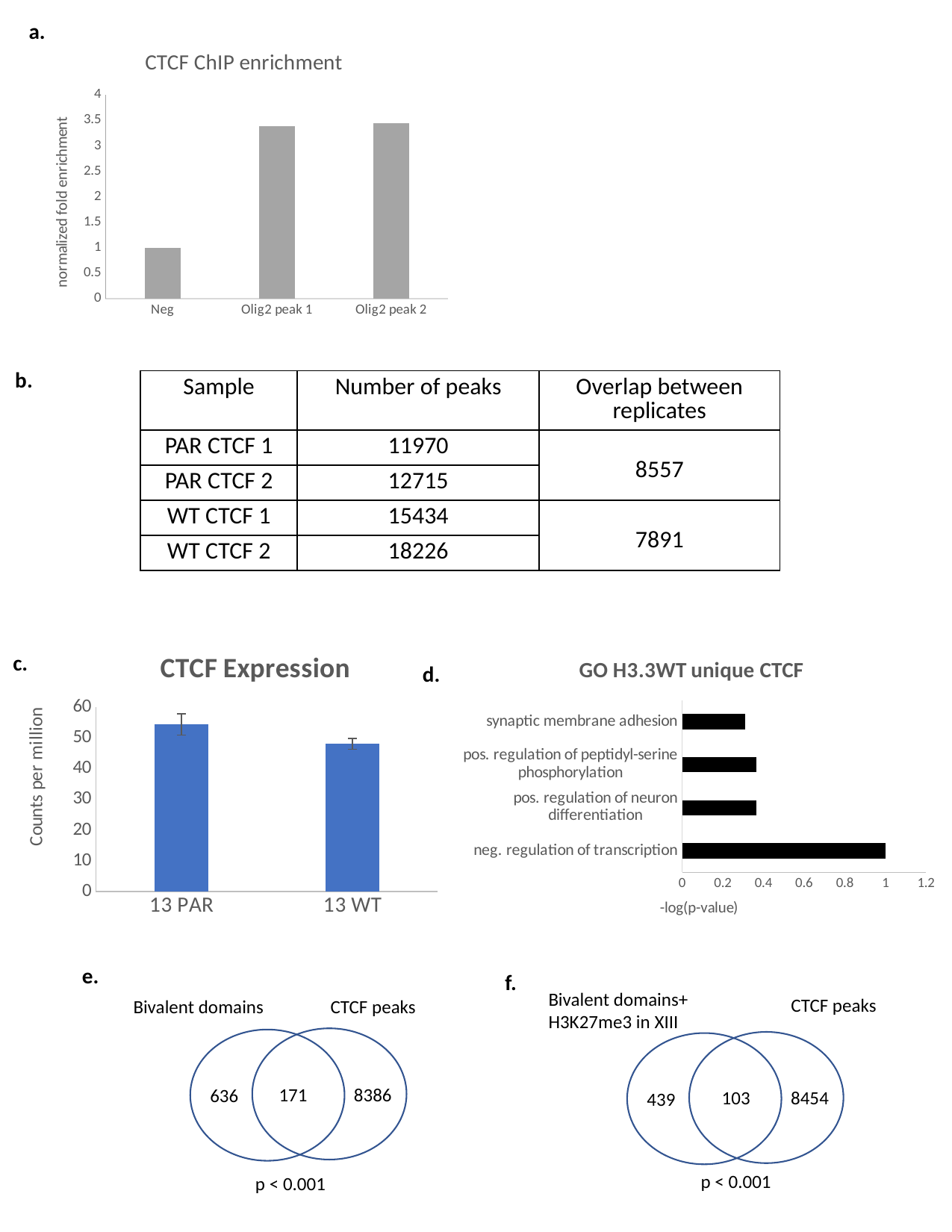

a.
### Chart: CTCF ChIP enrichment
| Category | |
|---|---|
| Neg | 1.0 |
| Olig2 peak 1 | 3.379164694620097 |
| Olig2 peak 2 | 3.4422057489773152 |b.
| Sample | Number of peaks | Overlap between replicates |
| --- | --- | --- |
| PAR CTCF 1 | 11970 | 8557 |
| PAR CTCF 2 | 12715 | |
| WT CTCF 1 | 15434 | 7891 |
| WT CTCF 2 | 18226 | |
### Chart: CTCF Expression
| Category | |
|---|---|
| 13 PAR | 54.35929753057655 |
| 13 WT | 48.036488013676546 |
### Chart: GO H3.3WT unique CTCF
| Category | |
|---|---|
| neg. regulation of transcription | 1.0 |
| pos. regulation of neuron differentiation | 0.36653154442041347 |
| pos. regulation of peptidyl-serine phosphorylation | 0.36653154442041347 |
| synaptic membrane adhesion | 0.3098039199714863 |c.
d.
e.
f.
Bivalent domains+
H3K27me3 in XIII
CTCF peaks
Bivalent domains
CTCF peaks
171
8386
636
103
8454
439
p < 0.001
p < 0.001

## Slide 7
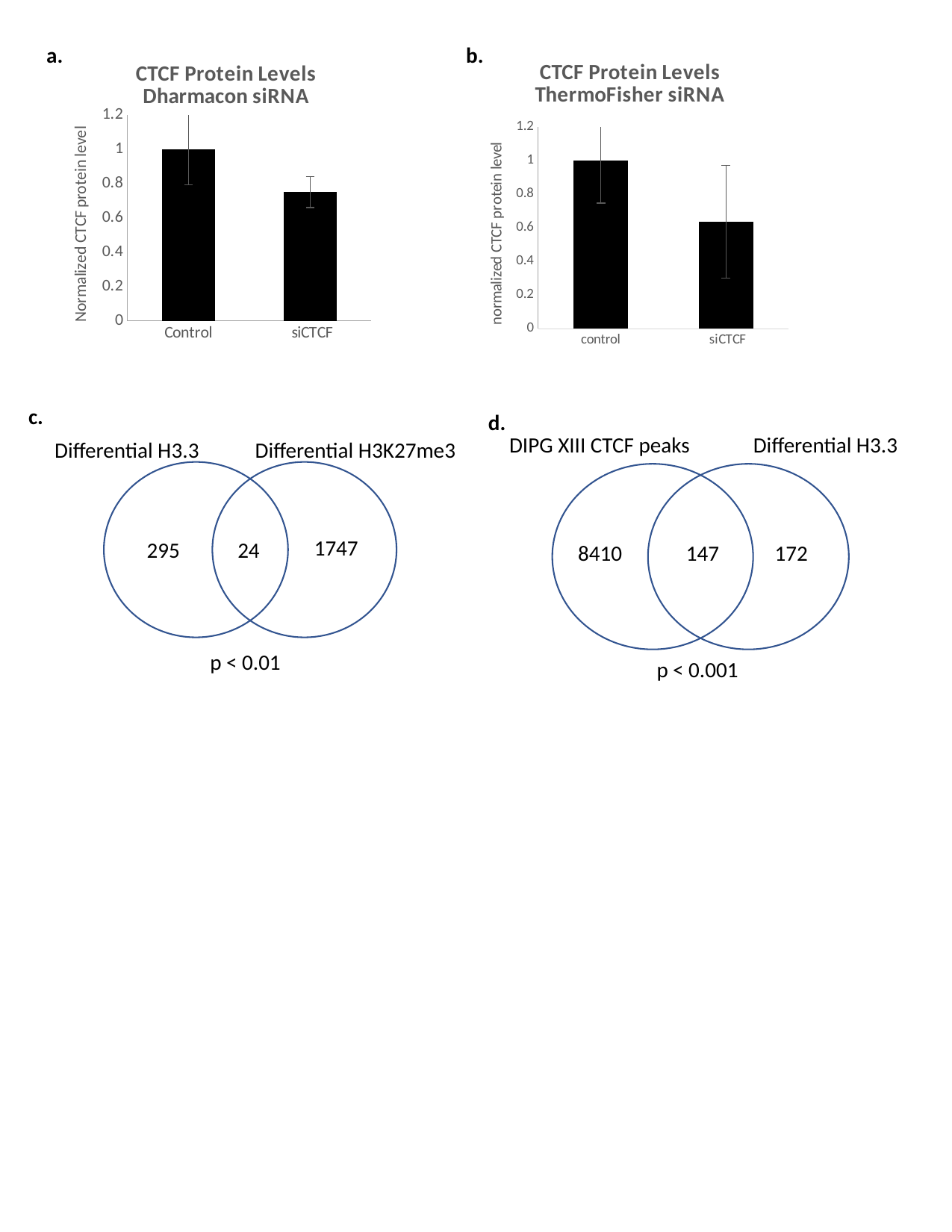

a.
b.
### Chart: CTCF Protein Levels ThermoFisher siRNA
| Category | |
|---|---|
| control | 1.0000000000000002 |
| siCTCF | 0.6369884245521268 |
### Chart: CTCF Protein Levels Dharmacon siRNA
| Category | |
|---|---|
| Control | 0.9999999999999999 |
| siCTCF | 0.7496942745538719 |c.
d.
DIPG XIII CTCF peaks
Differential H3.3
Differential H3K27me3
Differential H3.3
1747
295
24
8410
147
172
p < 0.01
p < 0.001

## Slide 8
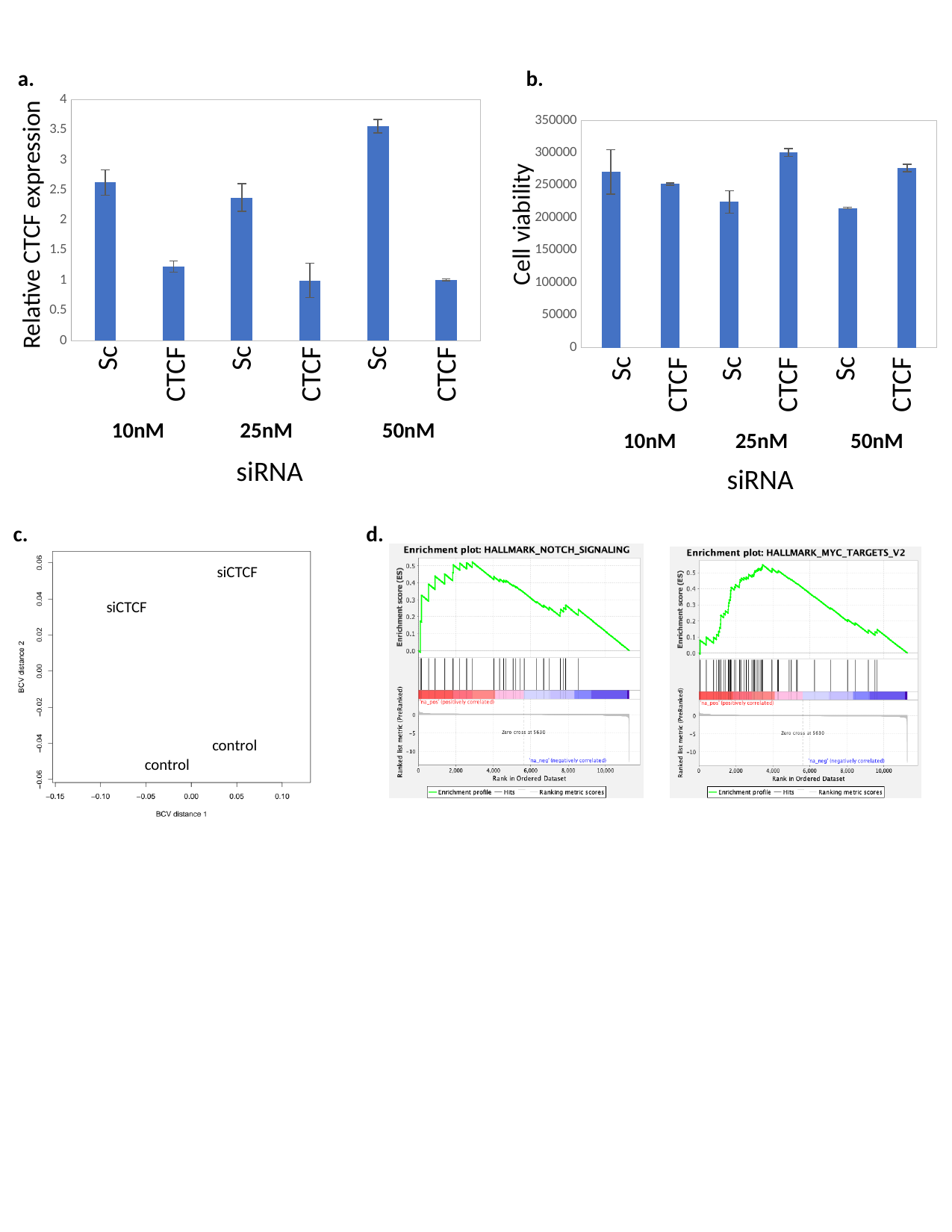

a.
b.
### Chart
| Category | |
|---|---|
| 13PAR (-) 10nM | 2.6268491117568122 |
| 13PAR CTCF 10nM | 1.231144413344917 |
| 13PAR (-) 25nM | 2.378414230005445 |
| 13PAR CTCF 25nM | 1.0 |
| 13PAR (-) 50nM | 3.5635948725613584 |
| 13PAR CTCF 25nM | 1.0092848012118731 |
### Chart
| Category | |
|---|---|
| 13PAR(-) 10 | 270700.3333333333 |
| 13PAR CTCF10 | 252307.66666666666 |
| 13PAR(-) 25 | 224536.0 |
| 13PAR CTCF25 | 300641.6666666667 |
| 13PAR(-) 50 | 215213.33333333334 |
| 13PAR CTCF 50 | 276941.0 |Cell viability
Relative CTCF expression
Sc
Sc
Sc
Sc
Sc
Sc
CTCF
CTCF
CTCF
CTCF
CTCF
CTCF
10nM
25nM
50nM
10nM
25nM
50nM
siRNA
siRNA
c.
d.
siCTCF
siCTCF
control
control

## Slide 9
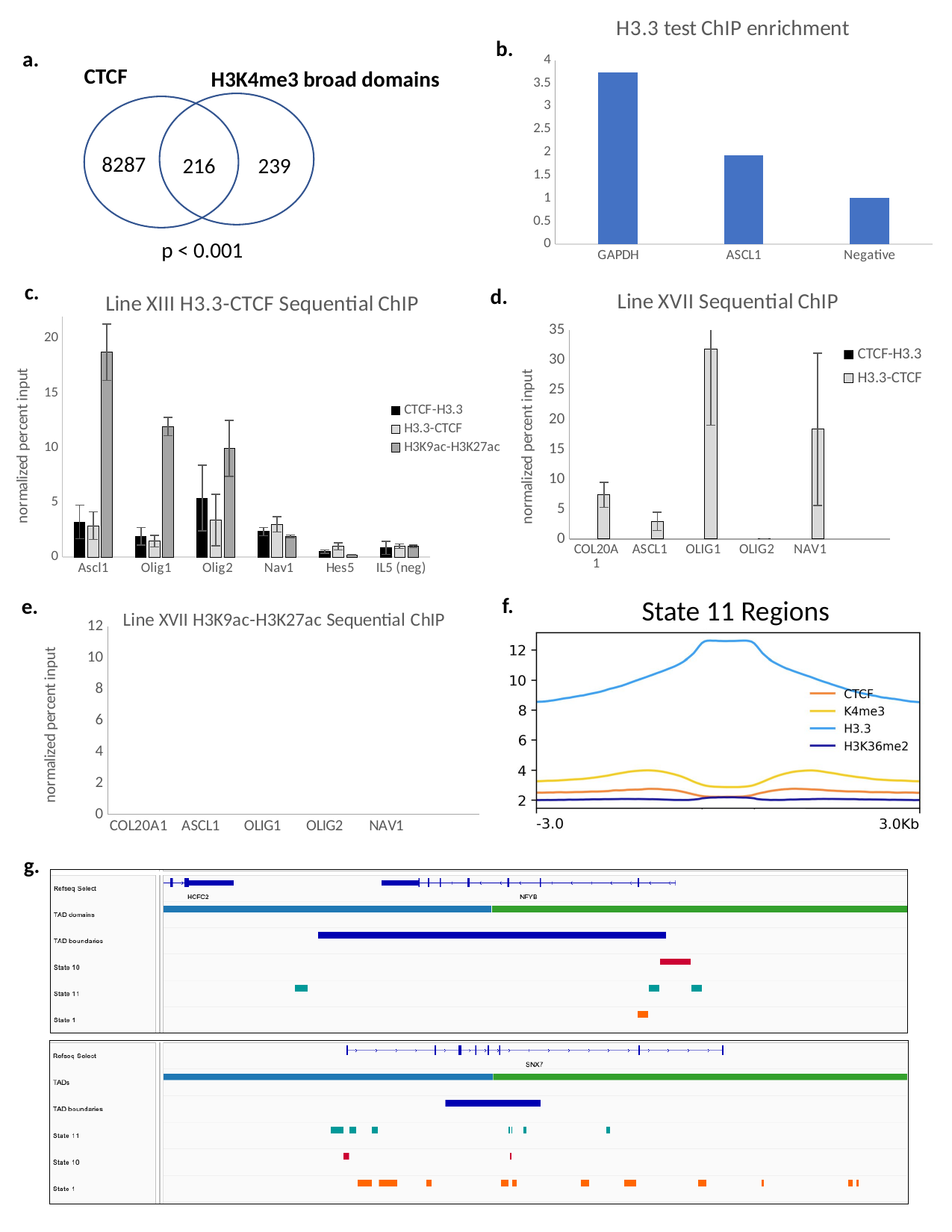

### Chart: H3.3 test ChIP enrichment
| Category | |
|---|---|
| GAPDH | 3.7321319661472274 |
| ASCL1 | 1.936341391965765 |
| Negative | 1.0 |b.
a.
CTCF
H3K4me3 broad domains
8287
216
239
p < 0.001
c.
### Chart: Line XVII Sequential ChIP
| Category | | |
|---|---|---|
| COL20A1 | 2.947775054244341 | 7.38681632285356 |
| ASCL1 | 1.5052467474110656 | 2.9271700885933662 |
| OLIG1 | 5.009808835478478 | 31.785371601884787 |
| OLIG2 | 0.066523136403335 | 0.03918872050226501 |
| NAV1 | 11.84711422366196 | 18.379225120615605 |
### Chart: Line XIII H3.3-CTCF Sequential ChIP
| Category | | | |
|---|---|---|---|
| Ascl1 | 3.2157620330426018 | 2.8696041738208806 | 18.739015019817284 |
| Olig1 | 1.9068110066940298 | 1.4619800309201556 | 11.938089660374226 |
| Olig2 | 5.398810096696667 | 3.378441916718581 | 9.9454049068056 |
| Nav1 | 2.333886045428781 | 2.995915629516903 | 1.9008386556014572 |
| Hes5 | 0.4974615939769891 | 0.9836144911927237 | 0.20105500245584976 |
| IL5 (neg) | 0.8446755050379352 | 1.0 | 1.0 |d.
f.
e.
State 11 Regions
### Chart: Line XVII H3K9ac-H3K27ac Sequential ChIP
| Category | |
|---|---|
| COL20A1 | 48.869629456285544 |
| ASCL1 | 361.7111148323936 |
| OLIG1 | 4824.853042106276 |
| OLIG2 | 17.805782003184706 |
| NAV1 | 1839.3247418328426 |
g.
